# Improved space breakdown method – A robust clustering technique for spike sorting

**DOI:** 10.3389/fncom.2023.1019637

**Published:** 2023-02-20

**Authors:** Eugen-Richard Ardelean, Ana-Maria Ichim, Mihaela Dînşoreanu, Raul Cristian Mureşan

**Affiliations:** ^1^Department of Experimental and Theoretical Neuroscience, Transylvanian Institute of Neuroscience, Cluj-Napoca, Romania; ^2^Department of Computer Science, Technical University of Cluj-Napoca, Cluj-Napoca, Romania; ^3^STAR-UBB Institute, Babeş-Bolyai University, Cluj-Napoca, Romania

**Keywords:** clustering, density, grid, spike sorting, machine learning, overlapping clusters, different density

## Abstract

Space Breakdown Method (SBM) is a clustering algorithm that was developed specifically for low-dimensional neuronal spike sorting. Cluster overlap and imbalance are common characteristics of neuronal data that produce difficulties for clustering methods. SBM is able to identify overlapping clusters through its design of cluster centre identification and the expansion of these centres. SBM’s approach is to divide the distribution of values of each feature into chunks of equal size. In each of these chunks, the number of points is counted and based on this number the centres of clusters are found and expanded. SBM has been shown to be a contender for other well-known clustering algorithms especially for the particular case of two dimensions while being too computationally expensive for high-dimensional data. Here, we present two main improvements to the original algorithm in order to increase its ability to deal with high-dimensional data while preserving its performance: the initial array structure was substituted with a graph structure and the number of partitions has been made feature-dependent, denominating this improved version as the Improved Space Breakdown Method (ISBM). In addition, we propose a clustering validation metric that does not punish overclustering and such obtains more suitable evaluations of clustering for spike sorting. Extracellular data recorded from the brain is unlabelled, therefore we have chosen simulated neural data, to which we have the ground truth, to evaluate more accurately the performance. Evaluations conducted on synthetic data indicate that the proposed improvements reduce the space and time complexity of the original algorithm, while simultaneously leading to an increased performance on neural data when compared with other state-of-the-art algorithms.

**Code available at:**

https://github.com/ArdeleanRichard/Space-Breakdown-Method.

## 1. Introduction

### 1.1. Spike sorting

One of the most prominent techniques for recording the activity of the brain is extracellular electrophysiology (Carter and Shieh, 2015). The technique takes advantage of extracellular contacts, usually inserted into the cortex, which pick up the firing signals (spikes) of neighbouring neurons as well as the local-field potential. Since the spikes of many surrounding neurons are captured by the same recording electrode, the problem arises to identify which spike comes from which neuron. This is called spike sorting (Rey et al., 2015) and it can be more formally defined as the process of grouping spikes into clusters corresponding to their emitting neurons.

The main assumption behind spike sorting is that individual neurons tend to fire spikes of a similar waveform shape, but which are also different among different neurons. Nevertheless, under realistic conditions, the shape of a neuron’s spikes gets mixed with noise in the extracellular recording, and the spike can also exhibit some degree of variability (Rey et al., 2015). As a result, the spike shapes of that neuron form a cluster in a feature space and not a single point.

In the ideal case, each identified cluster corresponds to all and only the spikes of a neuron, recorded within a given amount of time. In reality, different phenomena can affect the data, leading to overlapping clusters. Moreover, the disparate firing rates of different neurons lead to clusters of different sizes, leading to an inherent imbalance in the data to be clustered. Furthermore, due to the fact that all neurons within a certain neighbourhood are recorded and that neurons fire at intervals of milliseconds, even a short time duration can result in a high volume of data.

The Spike Sorting procedure can be compartmentalised into a pipeline of four steps starting with the raw signal provided by the extracellular recording of neuronal activity to the final labelling of clusters (Quiroga, 2007). The steps have been delineated as follows: filtering, spike detection, feature extraction, and finally clustering. The filtering consists of applying a band-pass filter (usually 300–5,000 Hz) to limit the signal components to the frequency band corresponding to spikes. The next step is spike detection, which is most commonly done by thresholding by amplitude. This step is far from perfect because the choice of a threshold has to be performed by finding a trade-off between sensitivity and specificity. The third step consists of feature extraction and is used to extract the most informative features of the spikes and to reduce dimensionality in order to ease the workload of the clustering method. Features can be defined in many ways, and there is no single golden rule on how to achieve this (Mishra et al., 2017). Most commonly, features are extracted by applying principal component analysis (PCA) on the spike waveforms (Adamos et al., 2008). The last step of spike sorting is the clustering in the feature space, and this is the step that our study focuses on.

Importantly, the separability of the clusters is determined by the feature extraction step and is received by the clustering technique as an input. Here, we do not address the feature extraction step. In addition, because the clustering technique is a labelling tool, it will not modify the space it receives, but it will attempt to properly label it. For the case of spike sorting—where overlap is a constant difficulty—the quality of a clustering algorithm is given by its ability to identify overlap and assign as many samples as possible to the correct cluster.

Extracellular recording of neuronal spikes is by its nature a “blind” technique. Indeed, in the absence of other supplementary techniques, such as intracellular recording or optical imaging, it is impossible to determine with total objectivity what spike is generated by what neuron. Therefore, in extracellular data ground truth labels are absent. As a result, spike sorting of recorded neural data is an inherently unsupervised problem.

### 1.2. Space breakdown method

Space Breakdown Method (SBM) (Ardelean et al., 2019) was developed as a deterministic clustering algorithm specialised for spike sorting. It was designed to deal with some of the major characteristics of such data: imbalanced clusters, overlapping clusters, and high data volume. First, imbalanced clusters arise because neurons can have markedly different firing rates. For example, inhibitory fast spiking basket cells fire at much higher rates than their excitatory, pyramidal counterparts, even when they are directly neighbouring each other (Moca et al., 2014). This results in more discharges from some neurons than others, translating into clusters with more or less points, respectively. Second, overlapping clusters can appear due to phenomena such as electrode drift and similarity of features between spikes of different neurons (Lewicki, 1998). The input of the Clustering step is provided by the Feature Extraction step of Spike Sorting. Clustering does not inherently modify the data, it only assigns labels to each point of the dataset. It would be unrealistic to assume that Feature Extraction would produce perfectly separated clusters with no overlapping. Third, the large volume of data emerges because of multiple reasons. Brain tissue is dense, and a single electrode can register the action potentials of multiple neurons (Bear et al., 2015). Additionally, under stimulation some neurons can fire vigorously. Moreover, the recording duration usually spans tens of minutes or hours, which leads to putative observation of many spiking events. Consequently, in a recording of minutes, there is a possibility to observe thousands of spikes even on a single electrode. In addition, recent high-density probes, for example the Neuropixels (Jun et al., 2017), can exhibit hundreds to thousands of electrodes, yielding extremely large datasets. With recent developments, in both hardware and software, investigators have started using automated spike sorting pipelines, such as KiloSort (Pachitariu et al., 2016), that allow for the real time analysis of high-density probes. Moreover, the results of such pipelines can be inspected and curated, if necessary. Soon, if not already, manual sorting will become obsolete and automatic methods will become the standard.

The aim of SBM is to tackle the difficulties of neural data in order to correctly assign spikes to the neuron that produced them. Therefore, SBM operates under the assumption that the clusters have the characteristics of neural data, SBM’s mechanism identifies clusters as unimodal, as shown in Supplementary Figure A1. For spike sorting, overclustering (defining more clusters than the number of neurons), is more acceptable than the mixing of clusters because spikes from the same neuron, split into two or more clusters, can be manually (or automatically) joined in a later stage by merging the clusters. By contrast, mixing the spikes from multiple neurons in the same cluster usually makes it very hard, if not impossible, to segregate them later.

Taking these challenges into consideration, SBM was designed to have linear time complexity in relation to the number of samples in the dataset. This performance resulted in a compromise, as SBM has an exponential complexity of execution time and memory with the number of dimensions of the dataset. Our first aim was to improve the exponential complexity of the execution time and of memory of SBM without losing its accuracy. Our second goal was to improve the accuracy of the algorithm for datasets that present characteristics that are similar to those of neural data.

## 2. Materials and methods

### 2.1. State of the art methods

In this section, we will present a short description of a number of clustering algorithms along with the original SBM that will be used in the analysis, whereas a critical view of each will be presented in the discussions section. We have evaluated the original and improved version against classical and recent clustering algorithms. DBSCAN (Ester et al., 1996) and K-Means (MacQueen, 1967) are two commonly used classical clustering algorithms that have been used in spike sorting for a long time. K-Means has been first used for sorting spikes in 1988 (Salganicoff et al., 1988). MeanShift, Agglomerative Clustering, and Fuzzy C-Means (FCM) have also been used in spike sorting (Veerabhadrappa et al., 2020) and to evaluate the performance. Having such a diverse collection, we are able to compare our algorithm against partitional, hierarchical, and density-based clustering methods. Although K-Means has been around for a long time and can be in no way considered a recent algorithm, uses for it and variations of it can be found even in today’s tools and pipelines (Pachitariu et al., 2016; Caro-Martín et al., 2018), which renders them viable candidates for comparative analysis. We compared the performance of these algorithms with the original SBM and with its improved version on multiple datasets using several clustering performance metrics.

K-Means (MacQueen, 1967) is a partition-based clustering algorithm that divides the space into k partitions, each point being assigned to the cluster with the nearest centroid. A disadvantage of K-Means is that it requires the number of clusters as a parameter. K-Means is not deterministic in its original design, but through optimisations it can become more stable. K-Means has a time complexity of *O(ndki)*, where *n* is the number of samples, *d* is the number of dimensions, *k* is the number of clusters given as input, and *i* is the number of iterations. Within spike sorting, where high overlap can appear, K-Means has trouble in separating such clusters and requires the number of clusters which can be difficult to estimate.

DBSCAN (Ester et al., 1996) is a clustering algorithm based on density. It defines clusters as regions with high densities and it labels low density regions as noise. An advantage of DBSCAN is that it does not require the number of clusters as a parameter. Moreover, it is able to identify clusters of arbitrary shapes, but it is unable to identify clusters with different densities. DBSCAN is deterministic with the exception of the “border points” (points at the edge of the cluster). DBSCAN has a time complexity of *O(n^2^)*, where *n* is the number of samples. Within spike sorting, where imbalance is given by the nature of neural activity, DBSCAN may have trouble identifying lower density clusters when high density clusters are present.

K-TOPS is a clustering algorithm (Caro-Martín et al., 2018) introduced in 2018, based on K-Means. K-TOPS relies on features based on the shape, phase, and distribution of spikes in order to achieve the clustering. In order to estimate the number of clusters for K-Means, in K-TOPS the number of clusters is varied from two to the square root of the number of spikes and is validated through the use of internal performance metrics, namely Davies-Bouldin, Sillhouette, and Dunn. The first two have also been used to evaluate our method. The final clustering is achieved through the use of template optimisation in the phase space. Overall, K-TOPS is more than a clustering algorithm as it implies the use of a specific set of features and a step of post-processing.

In Veerabhadrappa et al. (2020), the authors present a historical compendium of clustering algorithms used in spike sorting and evaluate their performance using external metrics. Their results show that ISO-SPLIT, a recently developed method, has the best performance for the datasets used. Nonetheless, K-Means, the oldest clustering algorithm used, places third out of 25 tested algorithms, while Agglomerative Clustering is the fifth, FCM the seventh, MeanShift the twelfth, and DBSCAN is the last.

MeanShift (Cheng, 1995) is a centroid-based clustering algorithm that finds clusters by updating centroid candidates to the mean of the points within their region and it is exclusively employed in spike sorting (Veerabhadrappa et al., 2020). As a post-processing step, it eliminates duplicate candidates to identify the final clusters. It does not require the number of clusters as input, which is an advantage. With regard to the complexity, it has a time complexity of *O(n^2^)*, where *n* is the number of samples. Within spike sorting, where overlapping clusters is common, MeanShift may undercluster and identify spikes from different neurons as a single cluster and as such as being produced by the same neuron.

Agglomerative Clustering (Ackermann et al., 2014) is a hierarchical clustering algorithm, more specifically it approaches the problem in a “bottom-up” manner. The algorithm starts by assigning each sample to an individual cluster, throughout the iterations clusters are merged based on a proximity matrix until a certain number of clusters are formed. The linkage method chosen is Ward, which analyses the variance of clusters instead of the distance directly. A disadvantage of the algorithm is that it requires the number of clusters as input. Agglomerative Clustering has a time complexity of *O(n^3^)* and a space complexity of *O(n^2^)*, where *n* is the number of samples. Within spike sorting, the elevated time complexity can become a problem with long recordings and the possibility of underclustering, due to overlap, is a problem for the correct identification of neuronal activity.

Fuzzy C-Means (Bezdek et al., 1984), in contrast to the other methods, is a soft-clustering algorithm where instead of receiving a label, a sample receives a probability and therefore a sample can be assigned to more than one cluster. The inner workings of the algorithm are similar to those of K-Means and just as its precursor it requires the number of clusters as an input. Being a successor of K-Means, it brings the same difficulties when applied in spike sorting. Variants of FCM are still being created improving its performance for specific tasks (Zhang et al., 2019) and it is still being used in a number of clustering applications, such as pipelines for image segmentation (Tang et al., 2020).

HDBSCAN (Campello et al., 2013) is an extension of the DBSCAN algorithm that modifies the algorithm from a density-based into a hierarchical type. Similar to the original, it identifies spikes as dense population among sparser ones and it classifies a subset of the samples as noise. Conceptually, the algorithm links points together as a weighted graph. An efficient implementation can use Prim’s greedy algorithm to build the minimum spanning tree that connects the points of the graph. The algorithm has one impactful parameter, the min cluster size that determines the minimum number of points needed by a group to be considered a cluster. The HDBSCAN algorithm has a space complexity of *O(dn)* and a time complexity of *O(n^2^)*, where *n* represents the number of samples and *d* the dimensionality of the dataset.

ISO-SPLIT is a clustering algorithm developed in 2015 that has been designed for spike sorting (Magland and Barnett, 2016, Veerabhadrappa et al., 2020). It is able to operate on an unknown number of clusters, assuming the unimodality of the clusters. Through repeated iterations, it establishes the unimodality by using isotonic regression. It has been shown to outperform classical algorithms such as K-Means of Gaussian Mixture Models (Magland and Barnett, 2016). Its advantages are that it can handle non-Gaussian clusters and that it requires no parametrisation. Moreover, in Veerabhadrappa et al. (2020), the authors proclaim it as the best algorithm for their tests.

By contrast to clustering, spike sorting algorithms are more complex techniques, where clustering is just one step in the sorting pipeline, which also includes, among others, spike detection, feature extraction, cluster merging, etc. Along the years, many spike sorting algorithms have been developed. Among the most prominent are KiloSort (Pachitariu et al., 2016), SpykingCircus, and WaveClus. KiloSort is an automated spike sorting pipeline that has the ability to analyse recordings of high-density electrodes in real-time and offers the possibility of human intervention through a manual user interface for post-processing curation (Pachitariu et al., 2016). The detection of spikes is made using template matching and spike prototypes are stored based on the L2 norm difference. KiloSort uses these spike templates to initialise a variant of K-Means that has a modified loss function, which is invariant to the amplitude changes of spikes (Pachitariu et al., 2016). Its main advantage is computational, through the use of mathematical models for the creation of spike templates. The introduction of template matching is the main feature of KiloSort, that substituting the spike detection and feature extraction steps of the classical spike sorting pipeline structure. Thus KiloSort would not be a fair candidate for comparison with our clustering algorithm as it contains other steps of the spike sorting pipeline besides clustering and as such it falls beyond the scope of this work.

### 2.2. Space breakdown method

**FIGURE 1:**
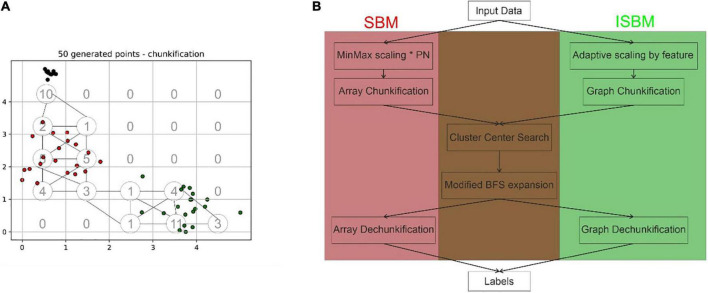
**(A)** Exemplification of the difference between the chunkification steps of the original SBM and the improved version, the space presented has been normalised, in the lower left corner of each grid cell is presented the corresponding value of the cell in the array structure, while the nodes and edges of the graph structure are indicated by circles and lines, respectively. In this case, the partitioning number is equal to 5. **(B)** Highlights the differences and commonalities between the original and improved versions of SBM based on the main processing steps.

Space Breakdown Method (Ardelean et al., 2019) is a grid-based clustering algorithm that divides the *N*-dimensional feature space through the use of a grid with equidistant grid lines. Following a min-max normalisation given by Equation 1, the algorithm will convert the sample space into an *N*-dimensional array, where each *N*-dimensional hypercube has an equivalent in a cell of said array, this is done through the grid. The min-max normalisation was chosen as it is easy to translate the points into a chosen range. The disadvantage of this normalisation is that it is sensitive to outliers. The addition of a pre-processing step of outlier detection and removal before applying the algorithm can be added in extreme cases. In each cell, the algorithm will store the number of points that can be found within the corresponding hypercube. An illustration of this conversion in a two-dimensional case can be found in Figure 1A, the values of chunks are shown in lower-left of each cell.


(1) $$n\,o\,r\,m\,a\,l\,i\,s\,e\,\left(X\right)=\frac{X-m\,i\,n\,\left(X\right)}{m\,a\,x\,\left(X\right)-m\,i\,n\,\left(X\right)}$$

The next step is the finding of centroid candidates, through the traversal of the array, these are cells that have values higher than all neighbours. Neighbours are defined as adjacent cells in the array, thus, cell (0, 0) is a neighbour of (0, 1), (1, 0), and (1, 1). Using Breadth-First Search (BFS) these centroid candidates are then expanded based on their neighbours to create the actual clusters. SBM through its design is able to surpass the limitations of other algorithms, such as K-Means’s inability to correctly identify overlapping clusters or DBSCAN’s tendency to assign noise to lower density clusters when high density clusters are present. It does not require the number of clusters as input as K-Means and other algorithms do and it has linear complexity with regard to the number of samples. However, it has an exponential complexity when it comes to the dimensionality of the data set.

A dataset is defined as having *n* samples in an *N*-dimensional space. The first step of SBM is the normalisation of every point in a range *[0, PN]* for all the *N* dimensions, where the partitioning number (*PN)* is a parameter. Each feature is divided into *PN* equal partitions, referred to as “*chunks*.” For a two-dimensional space, the chunks could be visualised as squares, while they would correspond to cubes for a three-dimensional space.

It can be inferred from the previous statement, that for an *N*-dimensional space, an *N*-dimensional array of size *PN^N^* is required to store the chunks. Each of these chunks will be represented in the array by the number of points contained by its interval. The process, which transforms the points of the dataset into an array representing the number of points in each interval, was named “chunkification.” Through the traversal of this array, the algorithm is searching for possible cluster centroids. The requirements of a chunk to become a cluster centroid are the following: to surpass the minimum threshold (another parameter) and to contain a larger number of points than its neighbours. The minimum threshold was added as a security measure for identifying conglomerations of isolated noise points as clusters. The local maxima are regarded as candidates for centroids of clusters in the dataset—nonetheless they may be merged later on, in the expansion step.

Once the candidates have been discovered, a BFS is applied on each centroid in order to expand the cluster to its neighbours. Through this process, chunks are receiving labels based on the cluster they have been assigned to. The labels are stored in an array of the same shape as the chunks. This requires another auxiliary array of size *PN^N^*. The last step is the assignment of labels to each point in the dataset. This step was named “dechunkification.” The translation is performed by identifying, for each point, the chunk it belongs to, and receiving the label of that chunk. Each point of the original dataset belongs to a chunk. To determine to which chunk a point belongs to, flooring of the point is applied.

The original SBM pipeline was modified to integrate the transition to the graph structure, this new structure allows for the reduction of spatial complexity which also results in a lessening of the processing time needed as fewer chunks need to be traversed. But the main steps of the algorithm have remained unchanged. The pseudocode for the improved SBM, modified to use a graph structure instead of the original array structure (Ardelean et al., 2019), is shown next:

1 SBM (dataset, PN, threshold)

2    X = normalise (dataset, PN)

3    graph = chunkification (X, PN)

4    ccs = findCentroids (graph, threshold)

5    for cc in ccs:

6     expand (graph, cc, label, ccs)

7    labels = dechunkification (graph, X)

8    return labels

To sum up, SBM consists of five sequential steps: *normalisation*, *chunkification*, *centroid search*, *expansion*, and *dechunkification*. The pseudocode of each step and the modifications made for the improvements can be found in Supplementary Appendix B. SBM has a time complexity of *O(n)* for the *normalise*, *chunkification*, and *dechunkification* algorithms, and *O(PN*^N^*)* for the *cluster centroid search* and *expansion* algorithms. Using the addition rule, due to the sequential application of these operations, the overall time complexity is *O(n + PN*^N^*).*

As the number of dimensions increases, not only does the time complexity increase, but also the space complexity, defined as the additional amount of memory required. As previously mentioned, the space complexity is exponential with regard to the number of dimensions *N*, and it is equal to *PN^N^*. This happens due to the required auxiliary *N*-dimensional array. Therefore, in certain cases of high dimensionality, a regular workstation may not be able to hold the amount of information needed for the auxiliary structure. An example was given in the discussion section of Ardelean et al. (2019): for a dataset with 1,000,000 points and 10 dimensions, even for a partition number as low as 5,5^10^ chunks are created of which the majority are empty. Empty chunks do not need to be stored because no operations are performed on them. Removal of these chunks when an array structure is used can be difficult without the additional traversal of those chunks which would increase the execution time.

### 2.3. The improved space breakdown method

#### 2.3.1. Solution overview

As mentioned before, a proportion of the chunks that are created will contain the value 0. These zero-valued chunks will not influence the result, but they will increase the complexity of the algorithm. By transitioning from an *N*-dimensional space to a graph, we can create nodes only for those chunks that have a value different from 0. Therefore, by using a graph structure, we limit the amount of chunks that can be created to the number of samples. This greatly reduces the amount of memory needed to run the algorithm for high dimensional datasets. A simple example of this trimming can be viewed in Figure 1, where the grid cells represent the cells of the array that would result from the original step of chunkification, while the circles indicate the nodes or chunks of the graph (the number of chunks for the improved version has been more than halved). Through this change of the data structure, the main logical steps of the algorithm do not need to be substituted in order to accommodate the modification.

The second improvement consists of modifying the partitioning number into a partitioning vector (PV). In the original version the chosen partitioning number was applied to all features (dimensions). This modification implies using the given partitioning number as the maximum number of partitions of a feature, in order to maintain the parameters of the original algorithm unchanged. The features that have the most information will retain the original partitioning number, while the other features will receive a partitioning number proportional to the information they contain. In this case, information is defined as variance.

#### 2.3.2. Detailed algorithm

The first improvement can be defined as the replacement of an *N*-dimensional array structure for a graph structure. This adaptation improves the space complexity of the algorithm and reduces the execution time for high-dimensional datasets. The first modification in the algorithm intervenes in the *chunkification* step. Therefore, the first step of the algorithm, that of *normalisation*, remains unchanged from the original version (Ardelean et al., 2019) and it consists of min-max normalisation of the input data *X*.

The normalisation is used to distribute the points within an interval that is easier to be split into chunks. With this in mind, the chosen technique was min-max normalisation as it allows for scaling afterward in an interval from 0 to a chosen number. Other techniques such as *Z*-score will produce different scales for each feature which would require an additional step of processing.

The graph structure is created as a *dictionary that* stores items in pairs of *key* and *value*. For our purposes, the *key* is a string value, while the *value* is another dictionary containing keys such as: *count*, *label*, and *visited*. At first, the graph is initialised as empty. Through the normalisation and rounding down step, the dataset shall have duplicates. Therefore, if a point has already been added as a node in the graph, we will increase its *count*. Otherwise, it will be added to the graph with a *count* of 1. By also initialising here the *label* of each node as 0, we remove the need of another graph to store the labels, as was the case for the original version of SBM that required another array. The *visited key* is used in the expansion step of the algorithm and is initialised to 0. The nodes of this graph are the equivalents of the chunks from the *N*-dimensional array that have a non-zero value, while the edges link two nodes that are immediate neighbours equivalent to the neighbouring cells from the previous array structure.

Through this change, the original required storage space of *O(PN*^N^*)* has been reduced to a maximum of the number of samples. Because we do not store nodes with a count of 0, we are able to reach the maximum only if each sample becomes its own node with a count of 1. Therefore, even in this particular case, the space complexity does not surpass the dataset size, being reduced to a maximum complexity of *O(n)*. This change also has an effect on the time complexity of the *centroid search*, *expansion.* In the original version, the *centroid search* had to traverse the whole space returned by the chunkification, which was *PN^N^*, while with the graph structure it would only need to check all the nodes created, denoted by *V*, where *V < n*. For the *expansion* step, because of the reduced number of neighbours of each chunk, where neighbours are found by using the edges of the graph, denoted by *E*, there is a reduction from *PN^N^* to (V + E), but the time complexity remains exponential, due to the exponentially increasing number of neighbours with the number of dimensions.

The second improvement is applied in the normalisation step and will not have an impact on the implementation of the following steps. This modification consists of an adaptive partitioning number. In the original version, the same PN was applied for all dimensions of the dataset. By changing the partitioning number for each dimension, the space complexity is further improved. Due to the fact that each dimension will have its own partitioning number, it may be called a PV that has the length equal to the number of dimensions of the dataset.

The required parameter of the algorithm remains the PN. However, it becomes the maximum number of partitions for a dimension. In order to identify the actual partitioning number of each dimension, the variance of each dimension is calculated by using normalised data, such that variances of the dimensions are comparable. The idea behind this modification is that a dimension with a lower variance will require a lower number of partitions to cluster correctly. In order to bring the variances into the range of PN, the variances are divided by the highest variance, bringing them in the [0, 1] interval. Then, by multiplying the variance array with PN, the variances are brought in the [0, PN] interval. In this way, the dimension with the highest variance receives the highest partitioning number and each dimension can have a different partitioning number. As previously mentioned, SBM’s original chunkification step could be visualised as partitioning the dataset into squares for two-dimensional and cubes for three-dimensional. Through this improvement, the chunkification step would partition the dataset into rectangles and cuboids for two-dimensional and three-dimensional, respectively. Through the min-max normalisation, the dataset is brought in the [0, 1] interval. Subsequently, the *N*-dimensional array of the dataset is multiplied with the PV of size N, thus each dimension being divided into its own number of partitions.

The labelling of SBM has been changed for the improvements. As a consequence, the improvements that constitute the Improved Space Breakdown Method are denoted as ISBM, have the clusters labelled starting from 0 as K-Means and DBSCAN have, while the noise is now labelled −1, just as DBSCAN. The original version of SBM had the clusters labelled starting from 1 and the noise as 0. In the figures presented, the noise will be coloured as grey for DBSCAN and ISBM and white for SBM.

We believe that noise points would be acceptable at least in two cases. First, for problems where one cannot recover the identity of all points due to the difficulty of assigning a border between clusters and where it is acceptable to lose a small fraction of the points (e.g., when one is more interested to find cluster centres for some *post hoc* computation, like data compression, etc.). The second case is when such noise points can be later manually curated by a user and assigned to a cluster or another based on some extra criteria, which are not apparent in the feature space. We now clarified this in the manuscript.

### 2.4. Clustering metrics

Multiple metrics were considered when analysing the performance of the clustering methods in order to have a robust view. These metrics are:

• Adjusted Rand Index (ARI) (Hubert and Arabie, 1985; Steinley, 2004; Vinh et al., 2010)
• Adjusted Mutual Information (AMI) (Strehl and Ghosh, 2002; Vinh et al., 2010)
• Purity (Manning et al., 2009)
• Fowlkes-Mallows Index (FMI) (Fowlkes and Mallows, 1983)
• V-Measure (VM) (Rosenberg and Hirschberg, 2007)

Adjusted Rand Index is based on the Rand Index (RI) metric with an added adjustment for chance. RI compares pairs of labels to see if they belong in the same cluster (called an agreement) or different clusters (called a disagreement) between the true and predicted labels. The clustering quality is given by the division of the agreements by the sum of the agreements and disagreements. ARI has a range of [−1, 1], where a score of 0 represents random assignment, a score of −1 represents independent labelling and a score of 1 represents the perfect match.

Adjusted Mutual Information is based on the Mutual Information (MI) metric with an added adjustment for chance. Moreover, the adjusted version of MI also has the normalisation step of Normalised Mutual Information (Vinh et al., 2009; Lazarenko and Bonald, 2021). MI is calculated between two clusters U and V using the count of their intersection, the count of each cluster and the total number of points in the dataset. AMI has a range of [−1, 1], where a score of 0 represents random assignment, a score of −1 represents independent label assignments and 1 represents the perfect labelling.

Purity is calculated as the sum of the maximum intersection between the true and predicted labels for a cluster divided by the number of samples. Purity has a range of [0, 1] where a perfect clustering has a value of 1. The disadvantage of Purity is that if each point is considered its own cluster, purity is 1. Thus, purity cannot estimate the correctness with regard to the number of clusters.

Fowlkes-Mallows Index is defined as the geometric mean of the pairwise precision and recall. Where precision is the number of true positives divided by the sum of true positives and false negatives, and recall is the number of true positives divided by the sum of true positives and false positives. FMI has a range of [0, 1], makes no assumption of cluster structure and random labelling receives a value of 0.

V-Measure is based on two other metrics, namely homogeneity and completeness. Both of these have a range between [0, 1]. VM is defined as their product divided by their sum with relation to a constant. VM also has a range of [0, 1] and makes no assumption of cluster structure, but random labelling will not yield zero scores for a high number of clusters.

### 2.5. Spike cluster score

In a preliminary analysis, the commonly used metrics, such as ARI and others, have been deemed unfitting for the evaluation of the accuracy for the following reasons. These metrics punish overclustering, which we considered acceptable within the context of spike sorting. The proposed metric is evaluated against classical clustering performance evaluation metrics in section 3.5. The metric developed can be categorised as an external metric and it takes as inputs the clustering labels and the ground truth. It represents the “purity” of the predicted label (*P*) with regard to the corresponding true label (*T*). For each unique true label *T*_*i*_, we can define the subset of the predicted labels corresponding to the subset of true labels with a value of *T*_*i*_ as *P(T_*i*_)*. In this subset of the predicted labels, we can calculate how many occurrences are of each label and we define the number of occurrences of a predicted label *j* in this subset as *count (P(T_*i*_) = P_*j*_)*. The score of the predicted labels for a unique true label is calculated as the division between the highest count of occurrences of a predicted label in the subset of true labels and the number of labels from the predicted labels that are equal to said predicted labels with the highest count. We can define the score of such a unique true label by the Equation 2. The overall score is calculated as the mean of all scores for all unique true labels.


(2) $$S\,c\,o\,r\,e\,\left(T\,i\right)=\frac{count\left(P\left(Ti\right)=Pj\right)}{count\left(P=Pj\right)},$$

where *P*_*j*_ is the predicted label with highest count.

Spike Cluster Score (SCS) is similar to the Purity metric. Like Purity, it will evaluate the clustering of each sample as its own cluster as a perfect clustering. SCS was developed to not punish overclustering, as overclustering is acceptable for some applications. We developed SCS instead of using Purity because we considered underclustering unacceptable and Purity did not penalise enough the underclustering of K-Means and DBSCAN. As a result, the score of Purity was similar across all algorithms. It is the user’s responsibility to evaluate how much overclustering is acceptable for each dataset. Clustering each sample as its own cluster will receive a perfect score but will not provide information. We recommend using SCS together with other performance evaluation metrics, while taking into consideration the weaknesses of each metric.

Moreover, SCS is unaffected by noise. Therefore, removing the points clustered as noise (for example, DBSCAN labels noise as −1, SBM as 0, and ISBM as −1) will not change the score. This is an advantage of SCS. For other metrics, it is necessary to first remove the samples labelled as noise to prevent contaminating their results.

### 2.6. Datasets

In the original study (Ardelean et al., 2019), SBM was evaluated for different types of datasets with different characteristics. Here, we used datasets that exhibit characteristics of neural data. The improvements added to the algorithm will be evaluated in comparison with the original version, and with K-Means, DBSCAN, MeanShift, Agglomerative Clustering, and FCM, on multiple datasets. In the following pages, we will present each of the datasets that will be used for the analysis.

#### 2.6.1. Unbalance-overlapping

The Unbalance-Overlapping (UO – Supplementary Figure A3a) dataset was generated to emulate the difficulties of neural data (Ardelean et al., 2019) and it was used to estimate the performance of the original version of SBM. It is a synthetic dataset with two dimensions containing 4,300 points divided into 6 clusters with Gaussian distributions with the following characteristics:

• Cluster of 500 points (red) with the centre at [−2,0]
• Cluster of 50 points (black) with the centre at [−2,3]
• Cluster of 1,000 points (yellow) with the centre at [3, −2]
• Cluster of 1,250 points (cyan) with the cluster centre at [5,6]
• Cluster of 250 points (blue) with the centre at [4, −1]
• Cluster of 1,250 points (green) with the centre at [1, −2]

#### 2.6.2. Simulations

Another batch of datasets (called simulations) for testing was generated by the Department of Engineering, University of Leicester, UK. The creation of these simulations was based on recordings from the monkey neocortex. The datasets contain 594 different spike shapes (Pedreira et al., 2012). For these datasets, the noise was considered to be a distinct cluster, named a multi-unit cluster. The amplitude of the spike of each cluster was modelled through random selection from a normal distribution (μ = 1.1, σ = 0.5) bounded within the 0.9–2.0 range. With the exception of the multi-unit cluster, which consists of 20 random spike shapes that have had the amplitude scaled to 0.5. Each spikes contains 79 samples that define its waveform. We used the following simulations for testing:

• Simulation 4 (Sim4 – Supplementary Figure A3b), containing 4 single-unit clusters and a multi-unit cluster (in total 5) with 5,127 points
• Simulation 1 (Sim1 – Supplementary Figure A3c), containing 16 single-unit clusters and a multi-unit cluster (in total 17) with 12,012 points
• Simulation 22 (Sim22 – Supplementary Figure A3d), containing 6 single-unit clusters and a multi-unit cluster (in total 7) with 7,101 points
• Simulation 21 (Sim21 – Supplementary Figure A3e), containing 4 single-unit cluster and a multi-unit cluster (in total 5) with 4,293 points
• Simulation 30 (Sim30 – Supplementary Figure A3f), containing 5 single-unit clusters and a multi-unit cluster (in total 6) with 5,210 points

The synthetic datasets have been generated such that no overlapping waveforms occur (Pedreira et al., 2012). This is a simplification of the classical data characteristics in spike sorting. Nevertheless, the authors (Pedreira et al., 2012) show that, even with such simplifications as a single multi-unit and no overlapping waveforms, no clustering algorithms were able to identify more than 8–10 clusters out of a maximum 20.

#### 2.6.3. Real data

*In vivo* electrophysiological data was recorded from the visual cortex of anaesthetised adult C57/Bl6 mice using A32-tet probes (NeuroNexus Technologies, Inc.) and 32-linear probes (Cambridge NeuroTech) at 32 kSamples/s (Multi Channel Systems MCS GmbH) during a visual perception task with moving stimuli. Visual stimuli consisted of full-field drifting gratings (0.11 cycles/deg; 1.75 cycles/s; variable contrast 25–100%; 8 directions in steps of 45°) presented monocularly on a Beetronics 12VG3 12-inch monitor with a resolution of 1,440 × 900, at 60 fps. All animals subjected to *in vivo* extracellular recording experiments were anaesthetised using isoflurane (5% for induction, 1–3% for maintenance) and placed in the stereotaxic holder (Stoelting Co., IL, United States). Heart rate, respiration rate, body temperature and pedal reflex were monitored throughout the experiment. Following a midline incision, a circular 1 mm craniotomy was performed over the left visual cortex of the animal (0–0.5 mm anterior to lambda, 2–2.5 mm lateral from midline). The multi-unit activity (MUA) was obtained by band-pass filtering the extracellular recorded data using a bidirectional Butterworth IIR filter, order 3 with cut-off frequencies between 300 Hz and 7 kHz. Subsequently, an amplitude threshold was calculated based on the standard deviation (SD) of the filtered signal and set at a factor of the SD [typically between 3 and 5 (Bârzan et al., 2020)]. All threshold crossings were identified as spikes and subsequently used as input for the feature extraction algorithm. To extract each spike, a window of 1.8 ms was extracted around threshold crossing (0.6 ms before and 1.2 ms after). At 32 kSamples/s this yielded 58 samples per spike. For each spike, these samples were used to create a two-dimensional feature space through PCA for the clustering algorithms.

To minimise animal use, multiple datasets were collected over 4–6 h from each animal. All experiments were performed in accordance with the European Communities Council Directive of 22 September 2010 (2010/63/EU) and approved by the Local Ethics Committee (approval 3/CE/02.11.2018), and by the National Sanitary and Veterinarian Authority (approval 147/04.12.2018).

## 3. Results

### 3.1. Parametrisation

The improvements of SBM, denoted as ISBM, were compared with the original version of SBM and with DBSCAN and K-Means on multiple datasets. SBM has already been established to have similar results to K-Means and DBSCAN on datasets that do not contain the characteristics of neural data. It has also been shown that it has a better performance on datasets with these characteristics (Ardelean et al., 2019).

The parameters for the clustering algorithms have been chosen in order to fit each dataset. For K-Means, the *k* parameter will be equal to the number of clusters in the dataset, equivalently for Agglomerative Clustering and FCM. In our experiments, we have found that the ward linkage of the Agglomerative Clustering gives the best results. For DBSCAN, the *eps* parameter was set using the elbow method for each dataset, while the *minsample* parameter was set to the logarithm of the number of samples. The eps parameter is quite sensitive to change, a higher value would result in underclustering, while a lower value in overclustering. To ensure the replicability of the results, we specify the parameters of each algorithm used for every dataset in Supplementary Table A1.

### 3.2. Evaluation of space complexity

One of the main concerns is the number of chunks. An evaluation of the number of chunks for a variable number of dimensions of the Sim4 dataset (containing 5,127 points) and for a partitioning number of 25 is presented in Table 1. The variable number of dimensions was obtained through the use of PCA (Mishra et al., 2017). Because the two improvements do not exclude each other, we will evaluate the algorithm containing both improvements as ISBM. As can be seen, the number of chunks increases exponentially with the number of dimensions for the original SBM, while it remains bounded for ISBM. In order to prove that both improvements have intrinsic value to the performance, Table 1 also includes the evaluation of number of chunks for only the graph structure improvement. The number of chunks, through the partitioning number, is part of the space and the time complexity equations, thus this cutback will improve performance.

**TABLE 1:** Number of chunks/Nodes evaluation – Sim4.

Number of dimensions/Features	SBM (array structure)	First improvement (graph structure)	ISBM (graph structure + adaptive partitioning)
2	625	343	188
3	15,625	1,659	321
4	390,625	4,072	532
5	9,765,625	4,981	744
6	244,140,625	5,111	988

There are a couple of factors that can indicate the choice of partitioning number. Intuitively, the partitioning number slices a feature, and the user is able to choose how fine or coarse this slicing is. The variance of the feature space and the number of points in the dataset indicate how dispersed the points are in that feature, thus we suggest the following formula to estimate a nearly optimal partitioning number:


$$P\,N=\frac{N*\text{max}\,\left(v\,a\,r\,i\,a\,n\,c\,e\,o\,f\,f\,e\,a\,t\,u\,r\,e\,s\right)}{10}$$

where, *PN* is the partitioning number and *N* is the number of samples in the dataset. The formula only uses the maximal variance of all features due to the second improvement brought to the algorithm that will change the partitioning number for the other features. By evaluating both synthetic and real datasets, we have found that the given formula can estimate the optimal partitioning number for some datasets, but only suboptimal for others. Thus, we recommend an exploratory search, starting from the partitioning number given by the formula.

### 3.3. Evaluation of time complexity

The second concern was the exponential time complexity in relation to the number of dimensions. This was evaluated on the Sim4 dataset and is shown in Table 2. A related inherent concern that validates the viability of the improvements is the evaluation of the linear time complexity in relation to the number of samples. This evaluation can be found in Table 3, and it is also illustrated in Supplementary Figure A2.

**TABLE 2:** Execution time (100 runs) by number of dimensions – Sim4.

Number of dimensions/ Features	K-Means (s)	DBSCAN (s)	MeanShift (s)	Agglomerative Clustering (s)	FCM (s)	HDBSCAN (s)	ISO-SPLIT (s)	SBM (s)	ISBM (s)
2	0.039	0.036	1.450	0.475	0.068	0.084	0.083	0.133	0.031
3	0.043	0.039	1.685	0.496	0.080	0.168	0.143	0.161	0.072
4	0.038	0.043	1.946	0.515	0.119	0.205	0.145	0.445	0.146
5	0.043	0.050	2.416	0.526	0.187	0.253	0.135	2.204	0.652
6	0.043	0.056	3.118	0.546	0.334	0.306	0.151	59.311	2.780

**TABLE 3:** Clustering execution time (100 runs) for varying the sample size of UO (two-dimensional synthetic dataset).

Number of samples	K-Means (s)	DBSCAN (s)	MeanShift (s)	Agglomerative Clustering (s)	FCM (s)	HDBSCAN (s)	ISO-SPLIT (s)	SBM (s)	ISBM (s)
4,300	0.048	0.068	1.650	0.254	0.204	0.066	0.65	0.079	0.038
8,600	0.054	0.174	2.154	1.183	0.384	0.143	0.78	0.119	0.060
12,900	0.079	0.300	2.389	2.930	0.636	0.221	0.103	0.184	0.079
17,200	0.091	0.485	2.636	5.133	0.952	0.345	0.141	0.227	0.102
21,500	0.107	0.686	2.728	7.970	1.224	0.496	0.156	0.263	0.119
25,800	0.124	0.873	2.911	12.075	1.637	0.622	0.198	0.305	0.138
30,100	0.155	1.153	3.295	16.499	2.012	0.705	0.236	0.361	0.165
34,400	0.166	1.564	3.613	21.577	2.323	0.798	0.278	0.412	0.187
38,700	0.200	1.783	4.040	29.510	2.537	0.947	0.312	0.441	0.205

**FIGURE 2:**
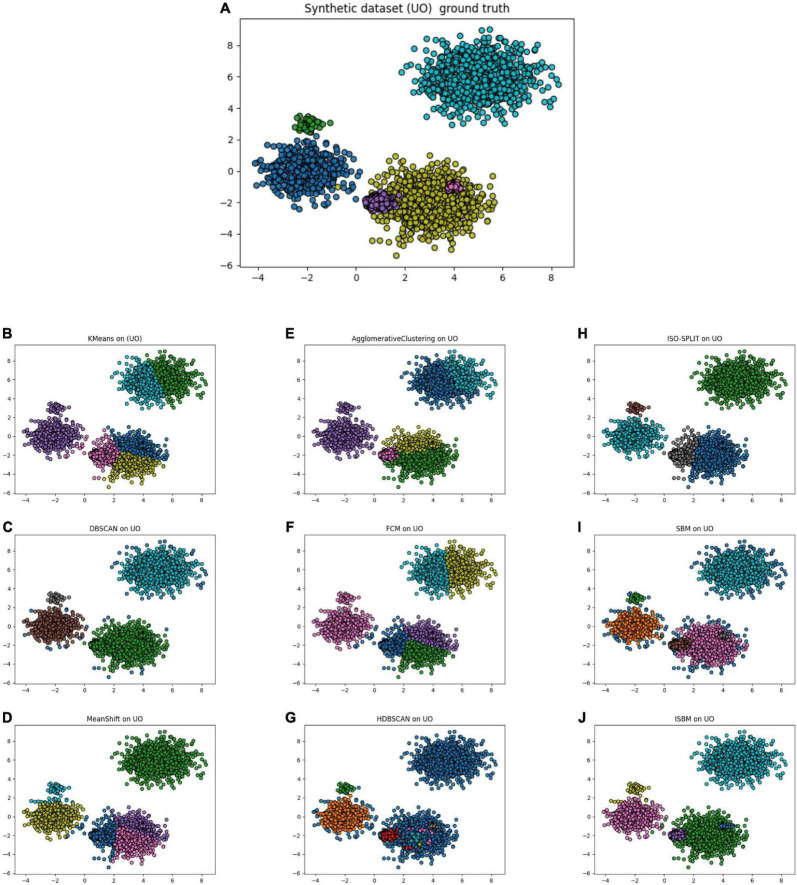
**(A)** Unbalance-Overlapping (UO), a synthetic dataset with its ground truth. **(B)** The result of K-Means on the UO dataset. **(C)** The result of DBSCAN on the UO dataset. **(D)** The result of MeanShift on the UO dataset. **(E)** The result of Agglomerative Clustering on the UO dataset. **(F)** The result of FCM on the UO dataset. **(G)** The result of HDBSCAN on the UO dataset. **(H)** The result of ISO-SPLIT on the UO dataset. **(I)** The result of the original version of SBM on the UO dataset. **(J)** The result of the improved SBM (ISBM) on the UO dataset.

Through these evaluations, we have demonstrated that the first improvement brings about a significant reduction of the time and space complexity. The second improvement further reduces the space complexity. Finally, none of the improvements squander the linear execution time of the original version in relation to the number of samples—they actually improve it. The results of all clustering algorithms on the UO dataset are presented in Figure 2, the colours indicate the cluster assignment of each algorithm. The corresponding display on the Sim4 dataset can be found in Supplementary Figure A4. In Figure 2D, the results of SBM are presented on the UO dataset. It can be observed that the colours used are different. Furthermore, in Figure 2F, it can be observed that ISBM does not overcluster the top-right cluster as SBM does.

We also evaluated the duration of the execution for each algorithm for the chosen datasets with the following results (average over 100 runs) presented in Table 4:

**TABLE 4:** Clustering execution time (100 runs) on all datasets (reduced to two dimensions using PCA).

Dataset	K-Means (s)	DBSCAN (s)	MeanShift (s)	Agglomerative Clustering (s)	FCM (s)	HDBSCAN (s)	ISO-SPLIT (s)	SBM (s)	ISBM (s)
UO	0.048	0.067	1.665	0.260	0.222	0.066	0.083	0.110	0.038
Sim4	0.038	0.036	1.452	0.466	0.089	0.084	0.117	0.132	0.054
Sim1	0.298	0.095	1.473	2.823	3.832	0.423	0.732	0.353	0.109
Sim22	0.061	0.053	1.463	0.906	0.200	0.357	0.175	0.310	0.068
Sim21	0.035	0.032	1.451	0.316	0.051	0.077	0.054	0.104	0.034
Sim30	0.072	0.121	1.513	0.478	0.145	0.101	0.132	0.207	0.050

The implementation was written in Python (version 3.7) with the following libraries: NumPy (version 1.21.4), matplotlib (version 3.5.0), sklearn (version 1.0.1) and pandas (version 1.3.4). All evaluations of the algorithms were run on a laptop with AMD Ryzen 9 5900HX at 3.30 GHz with 8 cores hyperthreaded, 32 GB of RAM at 3,200 MHz, 2 TB SSD.

### 3.4. Analysis of proposed metric

**FIGURE 3:**
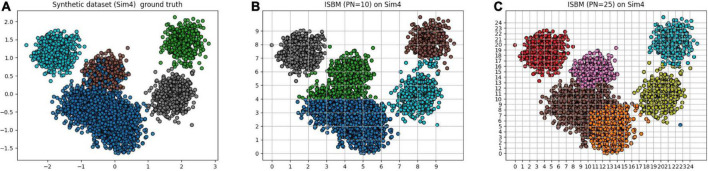
Simulation 4 (Sim4), a synthetic dataset reduced to two dimensions using PCA with its **(A)** ground truth. **(B)** The result of ISBM with PN = 10 on Sim4. **(C)** The result of ISBM with PN = 25 on Sim4.

A clear example of the punishment of overclustering by the performance metrics can be viewed in Table 5 and by comparing visually the results of Figure 3. In Figure 3A, the ground truth of the dataset is presented, while in Figure 3B ISBM has a PN of 10 and in Figure 3C, a value of 25. Most metrics will give a better result for PN = 10, even though the results of PN = 25 are more desirable within the context of spike sorting. This effect can be viewed through the performance evaluations presented in Table 5. The second reason for deeming classic performance metrics as unfit is that they also penalise algorithms that produce noise points such as DBSCAN or SBM, while noise points may also be acceptable for certain problems. Consequently, we have chosen to develop our own metric for the evaluation of the accuracy of results, termed as SCS. Nonetheless, we have not disregarded the performance estimation of the previously mentioned metrics and all the results can be found in section “3.5. Analysis of clustering metrics.” Due to the fact that the chosen metrics are bounded between [−1, 1] or [0, 1] range we have chosen to multiply them by 100 for easier visualisation of the performance.

**TABLE 5:** Metrics analysis on Sim4 (reduced to two dimensions using PCA).

Sim4	SBM	ISBM	SBM	ISBM
	PN = 10		PN = 25	
ARI	77.5	**80.2**	53.9	57.8
AMI	80.2	**85.0**	72.1	79.2
Purity	90.5	92.0	93.2	**96.5**
FMI	85.3	**87.1**	68.8	71.6
VM	80.2	**85.0**	72.1	79.2
**SCS**	89.7	89.8	93.8	**94.9**

Bolded values indicate the highest score obtained for each metric.

### 3.5. Analysis of clustering metrics

The evaluation of the performance of the algorithms with regard to the number of dimensions for the ARI, AMI, Purity, FMI, and VM metrics can be found in Table 6, the results on each dimension is separated by colour. The dataset has been reduced to the chosen dimensionality through PCA. The number of clusters of K-Means, Agglomerative Clustering, and FCM remains unchanged whilst varying the number of dimensions. The parametrisation of each clustering algorithm for every number of dimensions is shown in Supplementary Table 2.

**TABLE 6:** Clustering performance by number of dimensions – Sim4 (reduced to chosen dimensionality using PCA).

Dimensions	Metric	K-Means	DBSCAN	MeanShift	Agglomerative Clustering	FCM	HDBSCAN	ISO-SPLIT	SBM	ISBM
2	ARI	57.3	75.5	58.6	59.1	52.6	79.9	**91.7**	77.5	80.2
	AMI	79.6	77.9	80.5	77.7	73.4	87.0	**91.6**	80.2	85.0
	Purity	91.3	88.6	96.3	90.9	87.1	91.0	**96.9**	90.5	92.0
	FMI	71.1	85.2	72.3	72.4	67.6	88.5	**94.6**	84.9	87.1
	VM	79.6	77.9	80.5	77.8	73.5	87.0	**91.6**	83.8	85.0
	SCS	85.6	80.0	94.0	85.0	85.2	79.8	**94.9**	93.8	**94.9**
3	ARI	57.2	74.8	81.2	53.3	55.3	81.8	**99.5**	66.1	84.2
	AMI	79.4	82.5	86.1	78.3	77.9	82.0	**99.0**	69.4	87.6
	Purity	91.4	90.7	95.9	91.3	91.4	95.1	**99.8**	89.2	93.9
	FMI	71.0	84.8	87.8	68.3	69.7	88.2	**99.7**	80.7	89.7
	VM	79.5	82.5	86.1	78.3	77.9	82.1	**99.0**	73.4	87.6
	SCS	85.5	30.9	98.9	84.6	64.5	**99.8**	99.6	87.1	91.4
4	ARI	56.8	75.4	83.1	59.2	53.3	69.2	**99.7**	49.4	80.3
	AMI	79.5	80.3	87.1	80.1	75.0	78.0	**99.2**	59.3	82.4
	Purity	91.4	82.6	96.7	91.3	88.4	90.9	**99.9**	84.1	90.6
	FMI	70.8	86.3	89.1	72.5	68.2	80.4	**99.8**	60.8	88.5
	VM	79.5	80.3	87.1	80.2	75.0	78.1	**99.2**	62.2	82.5
	SCS	85.5	20.0	93.7	85.9	63.6	79.9	**99.7**	52.6	76.7

Bolded values indicate the highest score obtained for each metric.

The performance evaluation of the clustering algorithms given by all metrics for each dataset is shown in Table 7, each distinct dataset is given a different colour in the table for ease of visualisation. The evaluation was done on all labels, including the prediction of the clustering methods, even of those points labelled as noise that will reduce the performance estimation. The Sim type datasets have been reduced to two dimensions using PCA (Mishra et al., 2017). It can be observed from these tables that the overall best performance, considering all metrics, for all datasets is obtained through ISBM with the exception of the Sim4 dataset on which ISO-SPLIT performs the best with ISBM as a close second. It is important to note that among the synthetic datasets used, Sim4 is the simplest, as it contains the lowest number of clusters and the lowest amount of overlap. For Sim1, K-Means has a better score on Purity than ISBM.

**TABLE 7:** Clustering analysis on all datasets (reduced to two dimensions using PCA).

Dataset	Metric	K-Means	DBSCAN	MeanShift	Agglomerative Clustering	FCM	HDBSCAN	ISO-SPLIT	SBM	ISBM
UO	ARI	66.2	56.7	81.0	76.3	66.7	88.3	83.3	83.5	**95.0**
	AMI	77.0	75.5	83.5	83.0	77.3	85.9	86.8	82.5	**92.7**
	Purity	88.4	70.7	89.0	91.9	88.5	97.0	89.9	92.9	**97.5**
	FMI	73.9	73.0	85.6	81.9	74.3	91.1	87.7	87.4	**96.2**
	VM	77.0	75.6	83.5	83.0	77.3	85.9	86.8	82.5	**92.8**
	SCS	71.1	66.6	84.4	73.6	71.5	96.6	80.8	**97.8**	95.2
Sim4	ARI	57.3	75.5	58.6	59.1	52.6	79.9	**91.7**	77.5	80.2
	AMI	79.6	77.9	80.5	77.7	73.4	87.0	**91.6**	80.2	85.0
	Purity	91.3	88.6	**96.3**	90.9	87.1	91.0	**96.9**	90.5	92.0
	FMI	71.1	85.2	72.3	72.4	67.6	88.5	**94.6**	85.3	87.1
	VM	79.6	77.9	80.5	77.8	73.5	87.0	**91.6**	80.2	85.0
	SCS	85.6	80.0	94.0	85.0	85.2	79.8	**94.9**	93.8	**94.9**
Sim1	ARI	50.4	5.3	26.2	47.4	47.6	15.0	23.4	41.2	**52.9**
	AMI	74.1	26.9	58.1	72.1	73.3	50.5	62.0	68.4	**75.4**
	Purity	**79.2**	34	51.3	76.7	**79.2**	44.5	55.6	72.3	75.1
	FMI	55.6	33.7	46.9	52.6	52.9	41.4	46.5	46.8	**57.4**
	VM	74.2	41,2	58.2	72.2	73.4	50.6	62.1	68.6	**75.5**
	SCS	73.1	20.6	32.1	67.2	69.7	29.2	40.2	69.5	**73.9**
Sim22	ARI	66.1	50.3	81.8	63.6	65.5	59.1	**90.1**	80.6	89.0
	AMI	81.2	56.5	84.3	79.6	81.1	72.8	**89.1**	77.9	85.7
	Purity	91.3	74.2	88.9	90.3	91.3	78.2	**91.4**	86.1	93.4
	FMI	73.7	61.4	86.0	71.8	73.3	72.4	**92.5**	85.0	91.6
	VM	81.3	57.0	84.3	79.6	81.1	72.8	**89.2**	77.9	85.8
	SCS	80.8	60.7	78.9	77.8	80.0	70.8	83.7	**95.5**	90.8
Sim21	ARI	49.9	90.2	96.4	57.7	37.1	93.6	**97.1**	86.5	97.6
	AMI	71.8	80.1	**93.1**	76.2	62.2	90.2	**93.1**	77.9	91.2
	Purity	97.2	93.1	98.8	97.8	95.3	98.9	97.5	95.7	**99.1**
	FMI	71.7	95.3	98.3	76.9	62.2	96.9	98.6	93.4	**98.9**
	VM	71.8	80.1	**93.1**	76.3	62.3	90.2	**93.1**	78.0	91.2
	SCS	95.1	56.1	97.1	79.7	78.5	99.6	79.2	**99.9**	98.5
Sim30	ARI	55.4	50.6	90.5	56.2	54.8	81.7	82.8	47.3	**95.7**
	AMI	77.0	61.1	89.8	78.3	76.5	85.0	88.2	69.9	**92.9**
	Purity	92.2	75.0	96.1	92.0	92.1	91.4	92.4	93.5	**97.8**
	FMI	69.1	73.6	93.8	69.8	68.7	88.7	89.8	63.4	**97.2**
	VM	77.1	61.2	89.9	78.3	76.6	85.0	88.2	70.0	**93.0**
	SCS	87.1	63.3	92.2	82.0	79.4	82.8	82.6	95.3	**96.7**

Bolded values indicate the highest score obtained for each metric.

The scores of the algorithms for the SCS metric are also shown in Table 7 for all the points of the dataset. For the UO dataset the scores of K-Means and DBSCAN are lower due to the overlap and the different densities of the clusters. It is also noticeable that the clusterings of K-Means and DBSCAN are incorrect (Figures 2B, C). For the other datasets, due to the overlapping of the clusters, DBSCAN is severely punished for underclustering.

**FIGURE 4:**
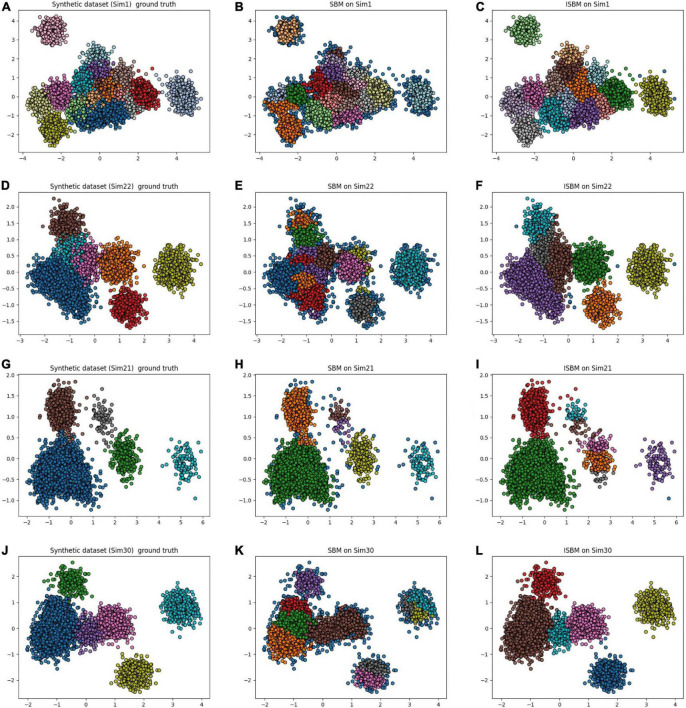
Original version and improved SBM comparison, the datasets were reduced to two dimensions using PCA. **(A)** Simulation 1 (Sim1), a synthetic dataset with its ground truth. **(B)** The result of the original SBM (PN = 46) on the Sim1 dataset. **(C)** The result of ISBM (PN = 46) on the Sim1 dataset. **(D)** Simulation 22 (Sim22), a synthetic dataset with its ground truth. **(E)** The result of the original SBM (PN = 46) on the Sim22 dataset. **(F)** The result of ISBM (PN = 46) on the Sim22 dataset. **(G)** Simulation 21 (Sim21), a synthetic dataset with its ground truth. **(H)** The result of the original SBM (PN = 20) on the Sim21 dataset. **(I)** The result of ISBM (PN = 20) on the Sim21 dataset. **(J)** Simulation 30 (Sim30), a synthetic dataset with its ground truth. **(K)** The result of the original SBM (PN = 40) on the Sim30 dataset. **(L)** The result of ISBM (PN = 40) on the Sim30 dataset.

An observation from Table 7 for SCS is that in some cases, the original version of SBM has a higher score than the improvement. This is true with regard to this metric, but as previously mentioned, SCS will not punish overclustering. For the “Sim” type datasets, the original version of SBM has a tendency to overcluster and therefore will receive a higher score. For these datasets, we have used PCA as the feature extraction method to reduce the dimensionality and it results in a high amount of overlap. By overclustering, there is a smaller chance to wrongly attribute spikes from multiple neurons to one cluster, but there is more work in the post-processing. In Figure 4, the results of the original version and the improvements of SBM are shown in relation to the ground truth. The overclustering of the original version of SBM can be clearly seen in Figures 4B, K and the fact that the improved SBM reproduces the ground truth labels more faithfully in Figures 4C, L.

The Sim1 dataset containing 17 clusters is the most complex of the chosen synthetic datasets. In Supplementary Figure A5, we show 3 of the clusters by their waveform in order to visualise the results of clustering methods. In Supplementary Figure A5a shows the ground truth and the correct separation of clusters and their relative waveforms. Supplementary Figure A5b the clustering of K-Means, Supplementary Figure A5c that of ISO-SPLIT, and Supplementary Figure A5d of ISBM.

### 3.6. Evaluation of performance on real data

**FIGURE 5:**
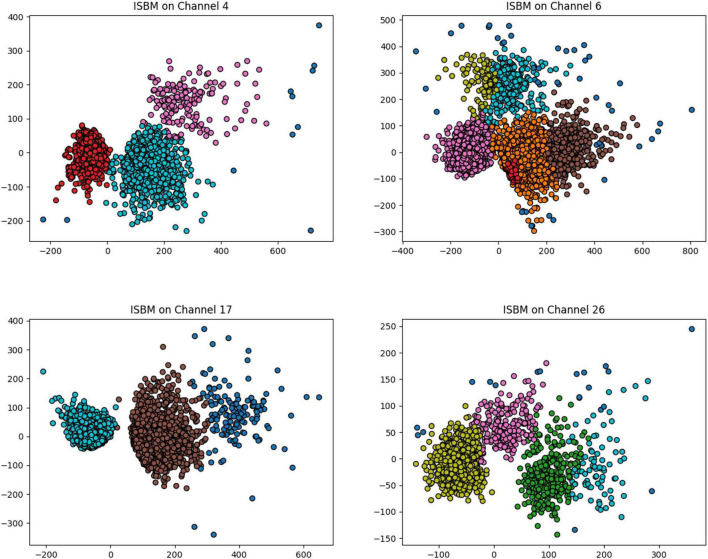
The result of improved SBM on four different selected channels of a real dataset recorded from the visual cortex of a mouse during a visual task. The probe used contained 32 single-channel electrodes.

The result of the improved SBM on 4 channels of a real dataset recorded using a 32 channels probe is shown in Figure 5. The four channels were chosen such that they have varying numbers of clusters and different distributions of clusters. In this figure, four plots are presented and each contains the samples registered by one electrode labelled by the algorithm. Because the data was recorded extracellularly, it contains no ground truth.

Channel 6 of this real dataset has the most complex distribution of clusters, in Supplementary Figure A6 we present the waveforms extracted by ISBM in comparison to those extracted by K-Means. The number of clusters of K-Means was chosen as 5 through the elbow method through the application of K-Means with various number of clusters.

**FIGURE 6:**
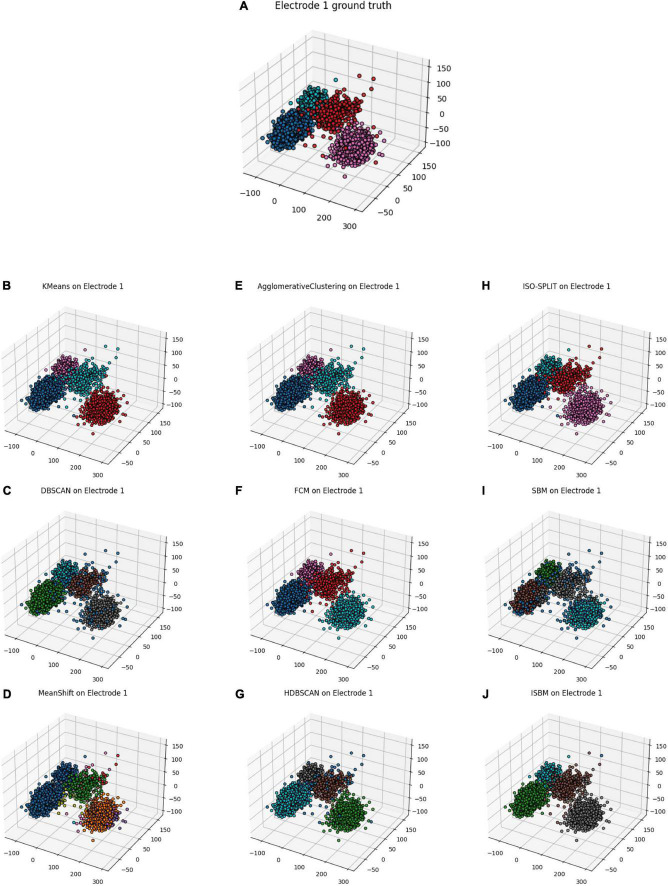
Real data from tetrode cleaned and sorted using K-Means with 4 clusters, electrode 1 has been selected as it offers the most separation. **(A)** Electrode 1 of the tetrode with the ground truth from the tetrode. **(B)** K-Means applied on electrode 1. **(C)** DBSCAN applied on electrode 1. **(D)** MeanShift applied on electrode 1. **(E)** Agglomerative Clustering applied on electrode 1. **(F)** FCM applied on electrode 1. **(G)** HDBSCAN applied on electrode 1. **(H)** ISO-SPLIT applied on electrode 1. **(I)** SBM applied on electrode 1. **(J)** ISBM applied on electrode 1.

Next, in order to gather sufficient information for establishing a “ground truth,” we have used a tetrode, whereby four electrodes are used to simultaneously record from a small region of extracellular space (Gray et al., 1995). After detecting each spike on the four electrodes, the amplitudes of the spike on the four channels were used to obtain a “ground truth.” The latter was obtained by using K-Means on the four-dimensional amplitude vectors. The resulting labels were taken as the “ground truth.” Subsequently, we tested the clustering algorithms by considering data from only one of the four electrodes. First, the dimensionality of the individual, single-electrode spikes was reduced to 3 by using PCA. Then, the clustering algorithms were applied, and results are shown in Figure 6. In Table 8, the results of the algorithms are evaluated through the use of performance metrics. As expected, K-Means has the best results as it was used to simulate the ground truth, with ISBM as a close second. We considered that the performance analysis with a generated ground truth is relevant to assess the correctness of the methods as it shows that the methods find similar clusters and are consistent. In a recent paper (Veerabhadrappa et al., 2020), the authors have evaluated the performance of 25 algorithms and K-Means placed third. Therefore, using K-Means as a next-to-best “ground truth” has justification.

**TABLE 8:** Metrics analysis.

	K-Means	DBSCAN	MeanShift	Agglomerative Clustering	FCM	HDBSCAN	ISO-SPLIT	SBM	ISBM	ISBM tetrode
ARI	98.8	97.0	97.7	98.4	53.7	98.0	98.9	49.3	98.2	94.6
AMI	96.3	90.5	91.1	95.8	70.8	92.9	96.6	64.0	93.9	92.7
Purity	99.3	97.8	99.0	99.2	91.5	98.8	99.3	85.9	98.7	98.1
FMI	99.4	98.4	98.7	99.1	71.9	98.9	99.4	84.0	99.0	97.0
VM	96.4	90.5	91.2	95.8	70.8	92.9	96.6	64.0	93.9	92.7
SCS	98.9	96.5	99.5	98.9	74.8	98.6	99.0	62.2	97.2	97.0

**FIGURE 7:**
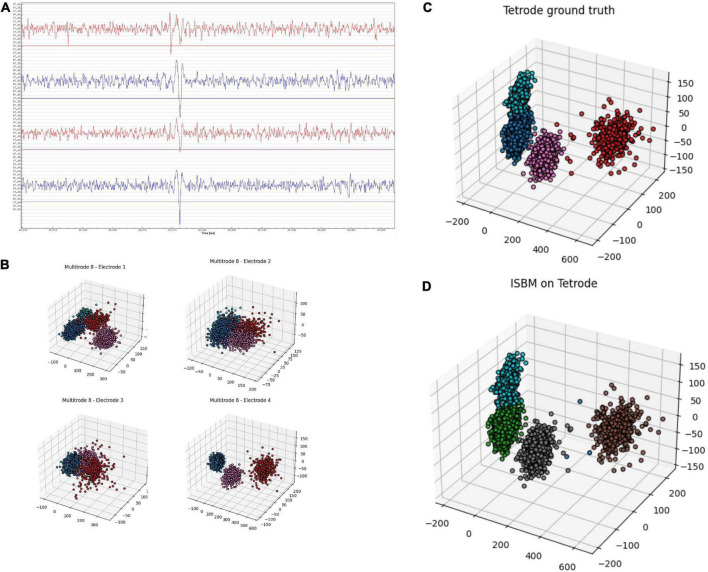
Tetrode analysis of ISBM, panel **(A)** shows a snippet of ∼50 ms of a tetrode recording from the mouse visual cortex during a visual stimuli presentation, panel **(B)** shows each of the four electrodes of the tetrode reduced to three dimensions by using PCA, the colours show a “ground truth” that was obtained by applying K-Means on the four-dimensional amplitudinal space, panel **(C)** presents the same “ground truth” on the space created by applying PCA on the whole tetrode, while panel **(D)** presents the labelling of ISBM on an eight-dimensional space extracted by PCA from the tetrode that is presented on the three-dimensional space for visualisation.

Tetrodes are able to capture spikes from different perspectives. Figure 7A shows a tetrode recording of the mouse visual cortex during a visual task. It is possible to use each channel separately, as shown in Figure 7B, where for each electrode we applied PCA to reduce the dimensionality to 2 and the colours represent a “ground truth” created through the use of K-Means on the four-dimensional space created by the aggregation of the amplitudes of spikes on each electrode. Figure 7B highlights the fact that even though the four tetrodes capture similar data, not every electrode is necessarily informative. In Figure 7C, we show the result of PCA on the data provided by the whole tetrode and the ground truth. It is worth noting that PCA applied electrode 1 shown in Figure 7B actually has more separation than the application of PCA on the tetrode. This happens due to the fact that the other electrodes are not as informative as electrode 1 and by combining their information, we actually lose separability. Nevertheless, we have applied ISBM on the space obtained by applying PCA on the whole tetrode domain. After reducing the dimensionality to 8, the performance evaluation is shown in the last column of Table 8 and in Figure 7D, where the labelling of ISBM is projected on the same space as Figure 7C.

## 4. Discussion and conclusion

Here, we introduced an improved version of the SBM clustering algorithm and investigated its performance by comparing it to the original version and other algorithms. Our focus was on neural data, which raises one of the most difficult problems, i.e., spike-sorting (Vinh et al., 2009). The algorithm was evaluated on both synthetic and real datasets. The synthetic datasets provide a ground truth that can be used to assess the performance of clustering algorithms. In this work (Pedreira et al., 2012), the creators of the dataset have also evaluated several clustering algorithms on the created datasets, showing that the best clustering algorithms were only able to identify from 8 to 10 clusters out of a maximum of 20 even though, from the perspective of spike sorting, the datasets are simple. Each dataset contains only one multi-unit cluster, and no overlapping waveforms transpire, whereas multi-unit clusters and overlapping waveforms can be a common occurrence in real data.

In this work, we have used PCA to extract the most important features and to reduce the dimensionality of the data. PCA is a well-established algorithm used as a reference for many spike sorting pipelines and is still commonly used inside and outside of spike sorting. This choice offers comparability with other methods present in the literature. Nevertheless, other feature extraction methods can be applied with any of the clustering algorithms and can be evaluated using various metrics, this falls outside of the aim of this analysis.

The well-known K-Means algorithm (MacQueen, 1967) is able to identify separated clusters with a high accuracy but it has difficulties with overlapping clusters. K-Means also requires the number of clusters as a parameter, which is difficult to provide for unlabelled data. DBSCAN (Ester et al., 1996), another widely employed density-based clustering technique, is able to identify separated clusters as well, provided that they have similar densities, but it tends to identify overlapping clusters as a single cluster, which is unacceptable in the context of spike sorting. MeanShift is able to model complex cluster with non-convex shapes without needing the number of clusters as an input, making it a highly viable candidate. However, MeanShift tends to be unable to differentiate between meaningful and meaningless modes and is unable to identify highly overlapping clusters. Agglomerative has a high complexity with regard to the number of samples making it hard to use in datasets of long recordings, also groups with close pairs, as is the case in overlapping clusters, may merge sooner than optimal resulting in the phenomenon of underclustering. FCM requires previous knowledge of the number of clusters as K-Means does which is a disadvantage within the scope of spike sorting, also similarly to K-Means it is unable to correctly separate clusters when overlapping clusters are present. By contrast, SBM (Ardelean et al., 2019) is able to identify overlapping clusters, provided that they exhibit a Gaussian distribution. This renders is particularly useful for the problem of spike sorting. SBM, like K-Means, scales linearly with the number of samples, but it has an exponential increase with regard to the number of dimensions. This renders SBM suboptimal for high-dimensional datasets due to memory and processing time concerns.

Through the improvements presented here, the space complexity of SBM has been significantly improved. Moreover, the processing time has been further reduced for high-dimensional data, allowing the algorithm to be used on high-dimensional datasets. With the addition of these improvements, the linear scalability of SBM with regard to the number of samples has not been changed and its performance on neural data has been increased, being able to outperform K-Means and DBSCAN on multiple datasets on almost all the metrics we have used.

The first improvement presented here tackles the space and time complexity regarding the number of dimensions, but without affecting the linear time complexity regarding the number of samples. The space complexity of SBM has been reduced from *O(PN*^N^*)* to only *O(n)*, where PN is the partitioning number, *N* is the number of dimensions, and *n* is the number of samples. Furthermore, the overall time complexity of the algorithm has been reduced to *O[n + (V + E)]*, where *n* is the number of samples, *V* is the number of nodes, and *E* is the number of edges between the nodes of the graph. This complexity is still exponential because, as the number of dimensions increases, the number of edges increases exponentially. However, this second improvement has achieved the goal of increasing the accuracy of the algorithm for neural data. Thus, we have improved the complexities of the algorithm while also increasing its accuracy on overlapping and imbalanced clusters.

With new developments of very high-density recording hardware, e.g., Neuropixels probes (Jun et al., 2017), the amount of neural data to be analysed may increase a 1000-fold. In addition, chronic, home-cage electrophysiological recordings, which typically track neural activity for days, are also generating increasingly larger datasets (Dhawale et al., 2017). Algorithms like DBSCAN will have reduced usability for such data, with a time complexity of *O(n^2^)* such that the processing of huge datasets will be much slower in comparison to algorithms with complexity *O(n)*, like K-Means and SBM. Furthermore, DBSCAN uses a distance matrix of size *n*^2^ and such that for large datasets it will require a huge amount of memory. By contrast, due to the linear scalability with regard to the number of samples and due to its reduced space complexity of *O(n)*, the improved version of SBM presented here may become a feasible choice for the spike-sorting of very large datasets by employing similar strategies to those of recently developed spike sorting pipelines, such as the subsampling of spikes and the clustering this subset in order to obtain a collection of templates based on the cluster centres. Furthermore, because of the shortcomings of K-Means in tackling overlapping clusters, we feel that ISBM may be used complementarily with K-Means if not even substitute it. From a theoretical perspective, ISBM may be able to substitute K-Means in newly developed spike sorters, such as KiloSort, since one issue that remains with such approaches is that overlaps can appear from the initial detection, and it requires an additional step of post-processing for removal (Pachitariu et al., 2016). We believe that ISBM can provide more performant clustering due to its ability to separate overlapping clusters based on unimodality.

To conclude, the changes made to the algorithm have improved its performance on all evaluated criteria, making it able to outperform other clustering techniques when applied to spike sorting. Moreover, they allow SBM to be used within the context of future updates to hardware and experimental designs, which will generate a significant increase in the amount of neural data to be analysed.

## Data availability statement

Publicly available datasets were analyzed in this study. These datasets were created by the Department of Engineering, University of Leicester, United Kingdom (Pedreira et al., 2012).

## Ethics statement

This animal study was reviewed and approved by the Local Ethics Committee (3/CE/02.11.2018) and the National Veterinary Authority (147/04.12.2018).

## Author contributions

E-RA: conceptualisation, software, writing—original draft, and visualisation. E-RA and RM: methodology. E-RA, MD, and RM: validation, formal analysis, and investigation. A-MI: data acquisition. E-RA and A-MI: data curation. E-RA, RM, MD, and A-MI: writing—review and editing. MD and RM: supervision and project administration. All authors contributed to the article and approved the submitted version.
