# Supplementary material for: Improved space breakdown method – A robust clustering technique for spike sorting
Source: Front Comput Neurosci. 2023 Feb 20;17:1019637. doi: 10.3389/fncom.2023.1019637 (PMC9986479; doi:10.3389/fncom.2023.1019637)
Supplement: Supplementary file 1 [file Data_Sheet_1.docx]

# Appendix A


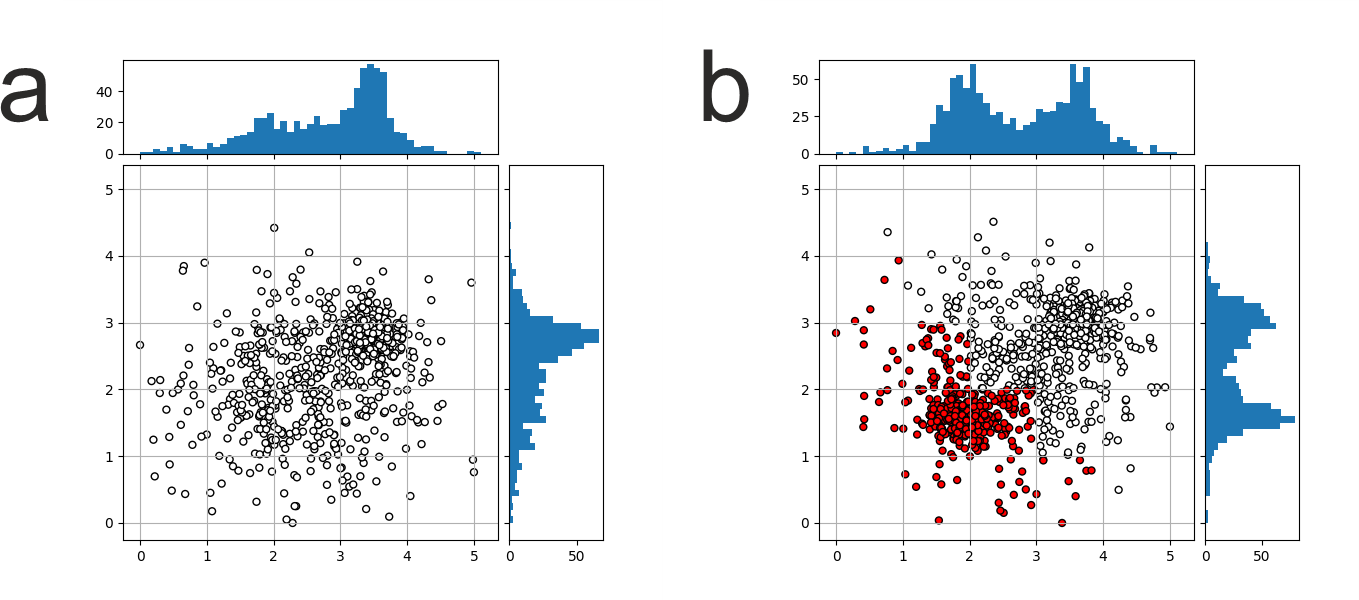


Figure 1 – SBM and ISBM’s results dependency on the modality of the data. Panel (a) shows a randomly generated unimodal cluster labelled as a single cluster (white) by SBM and ISBM, while (b) shows a bimodal cluster that is identified as two clusters (red and white) by SBM and ISBM.


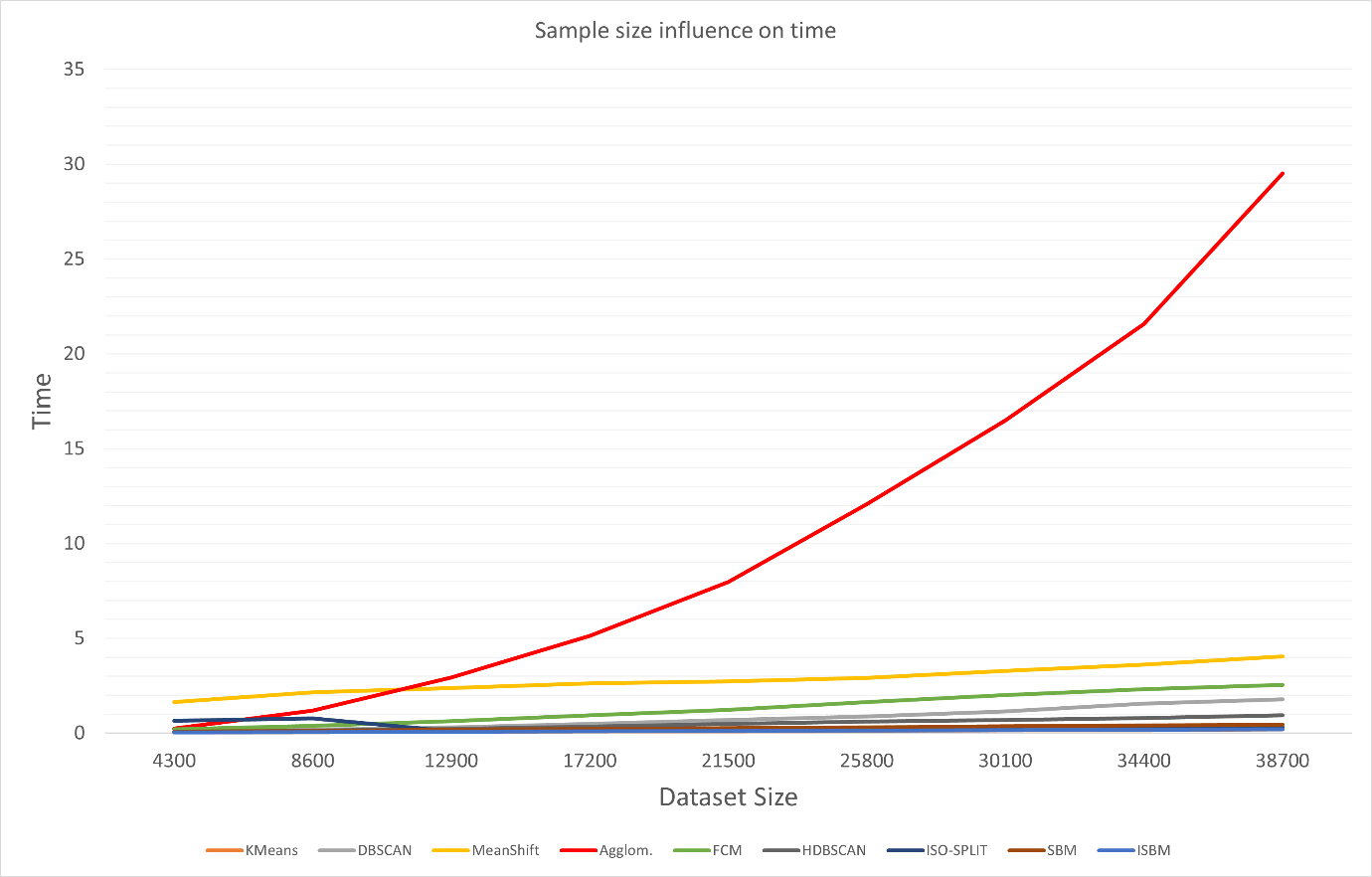


Figure 2 – Clustering execution time (100 runs) for varying sample size of the UO dataset, equivalent to Table III.


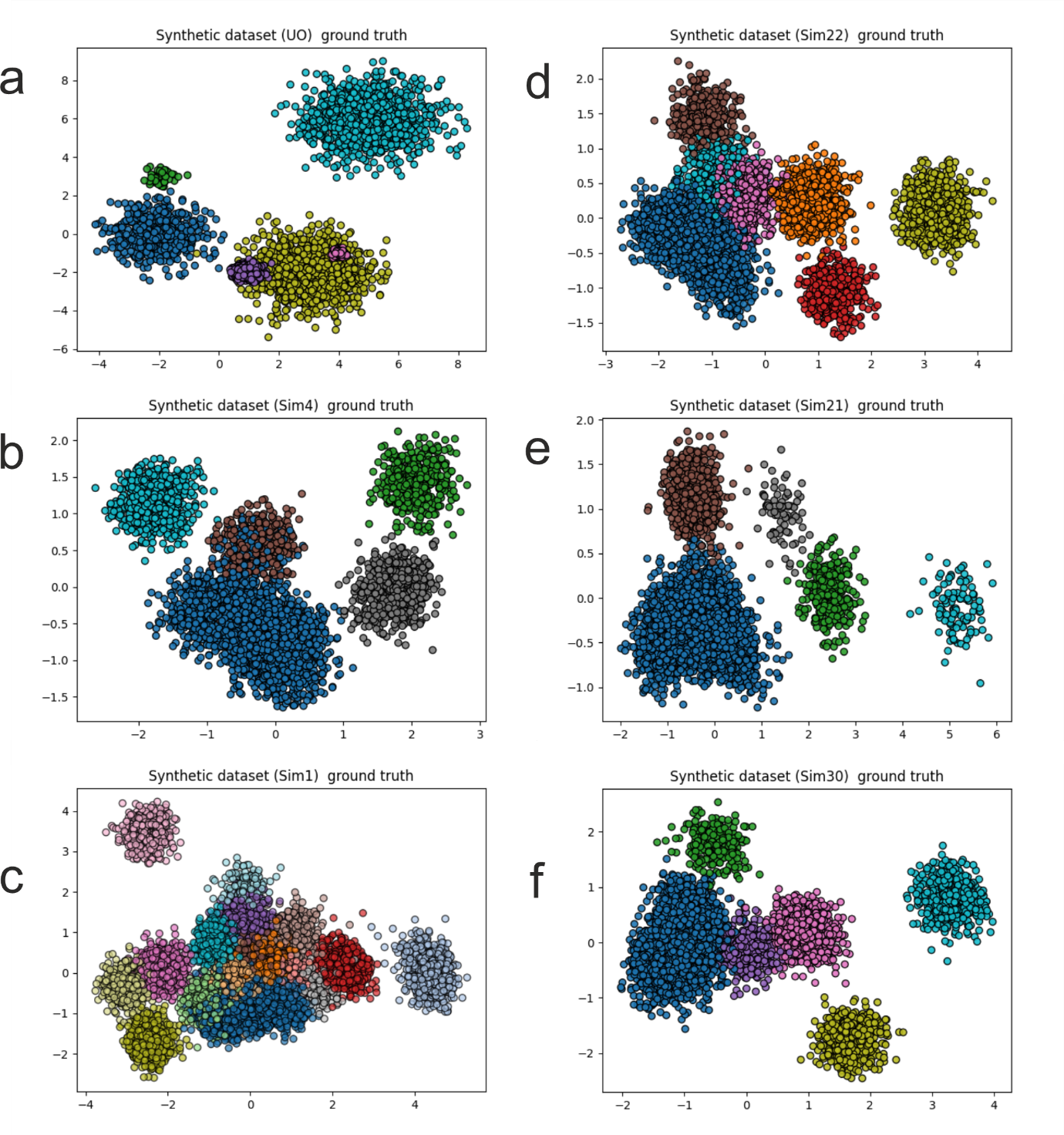


Figure 3 - Datasets *(a)*. Unbalance-Overlapping (UO), a synthetic dataset with its ground truth *(b)*. Simulation4 (Sim4), a synthetic dataset with its ground truth *(c)*. Simulation1 (Sim1), a synthetic dataset with its ground truth *(d)*. Simulation22 (Sim22), a synthetic dataset with its ground truth *(e)*. Simulation21 (Sim21), a synthetic dataset with its ground truth *(f)*. Simulation30 (Sim30), a synthetic dataset with its ground truth.


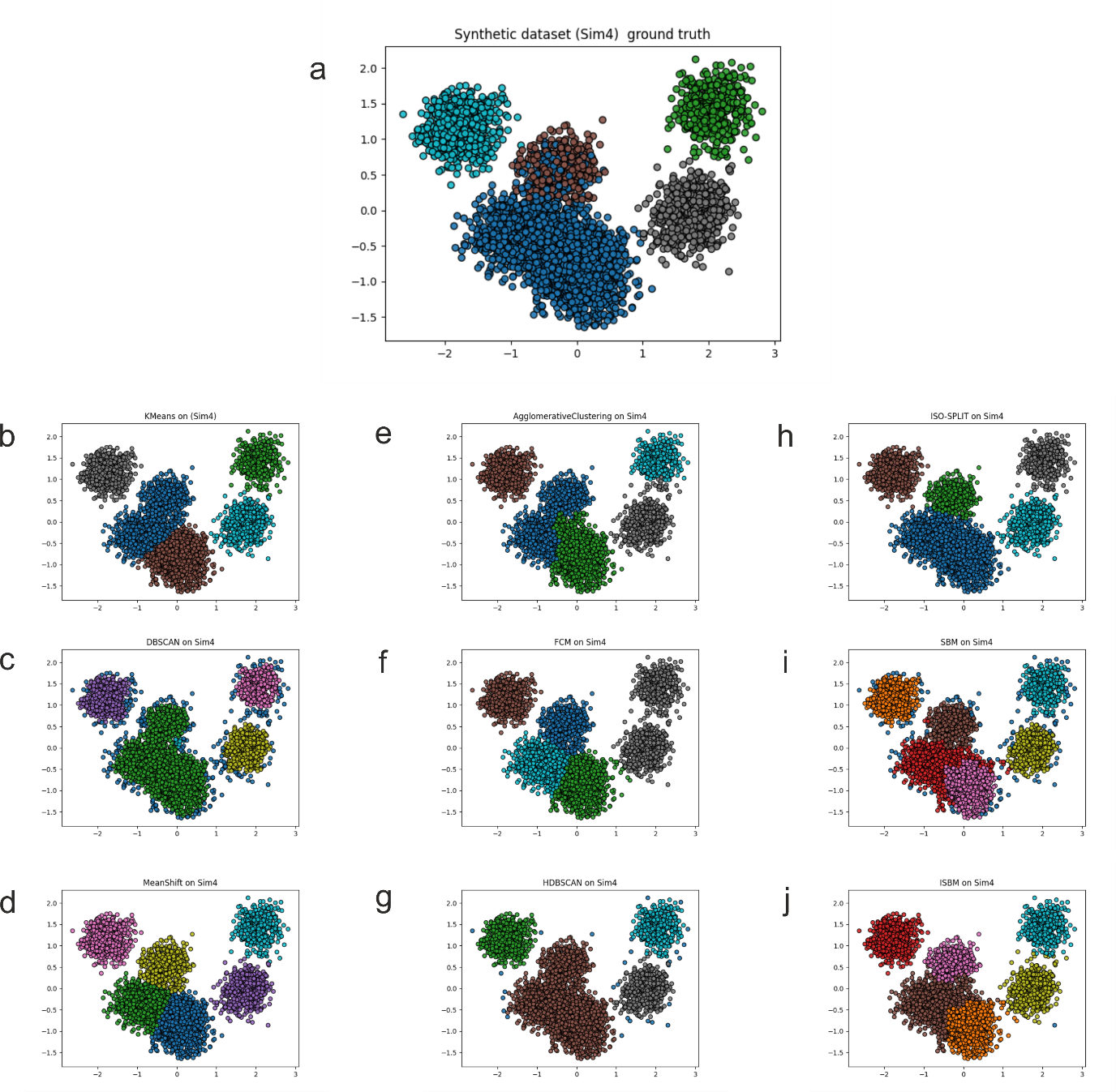


Figure 4 - *(a)*. Simulation 4 (Sim4), a synthetic dataset with its ground truth *(b)*. The result of K-Means on the Sim4 dataset *(c)*. The result of DBSCAN on the Sim4 dataset *(d)*. The result of MeanShift on the Sim4 dataset *(e)*. The result of Agglomerative Clustering on the Sim4 dataset *(f)*. The result of FCM on the Sim4 dataset *(g)* The result of HDBSCAN on the Sim4 dataset *(h)*. The result of the ISO-SPLIT on the Sim4 dataset *(i)*. The result of the original version of SBM on the Sim4 dataset *(j)*. The result of the improved SBM (ISBM) on the Sim4 dataset.


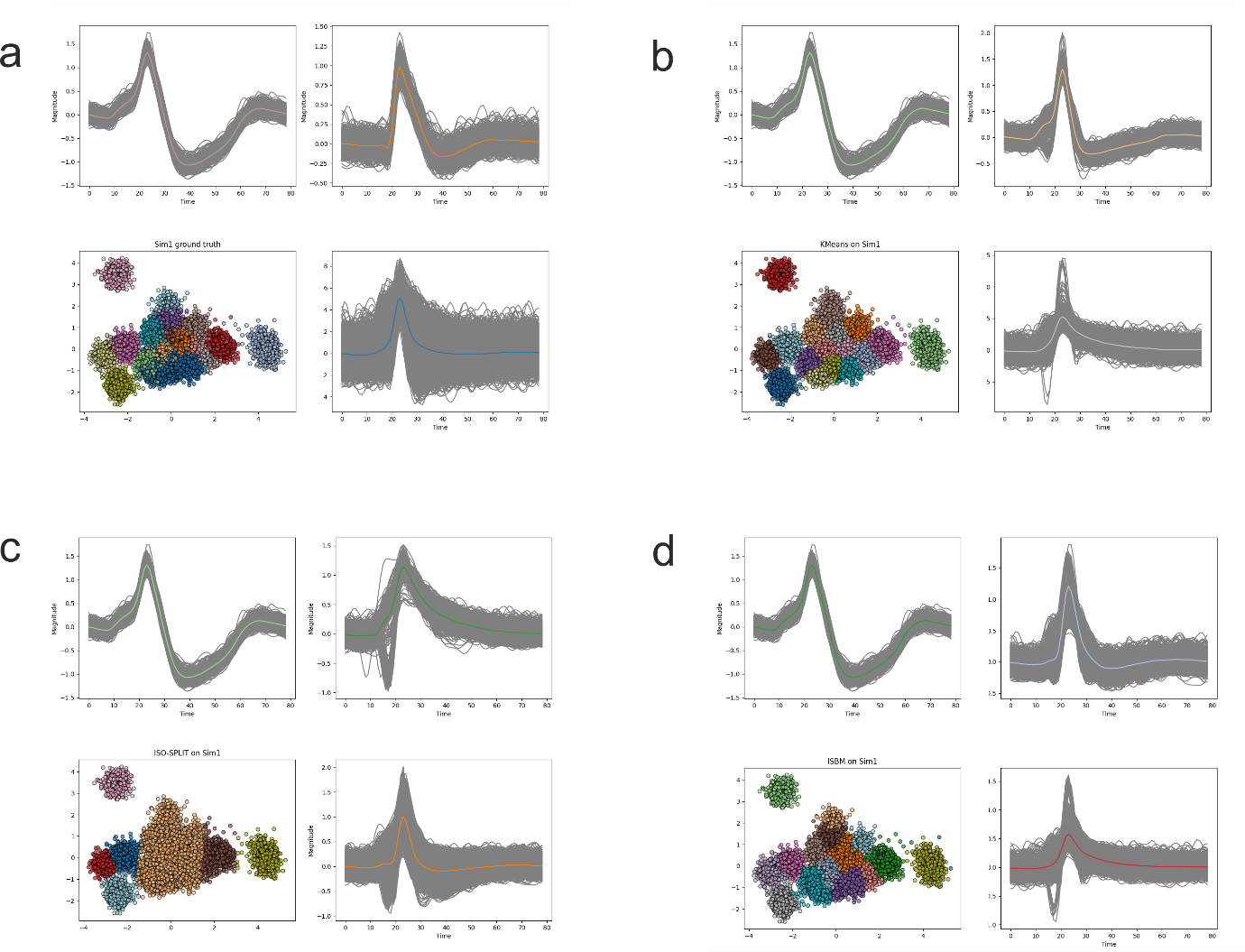


Figure 5 - Clustering of synthetic data and the visualization of the generated waveforms. All four panels respect the same format: the colored line represents both an identification of the cluster that contains those waveforms and the average spike of the cluster, the top-left figure shows the waveforms of the cluster found in the top-left, the top-right figure shows the waveforms of the cluster that has the most overlap with the blue cluster in the ground truth, the bottom-right figure shows the waveforms of the cluster that has the most overlap with the white cluster in the ground truth and the bottom-left figure shows (a) the ground truth in 2 dimensions (through PCA), (b) the clustering of K-Means, (c) the clustering of ISO-SPLIT and (d) the clustering of ISBM.


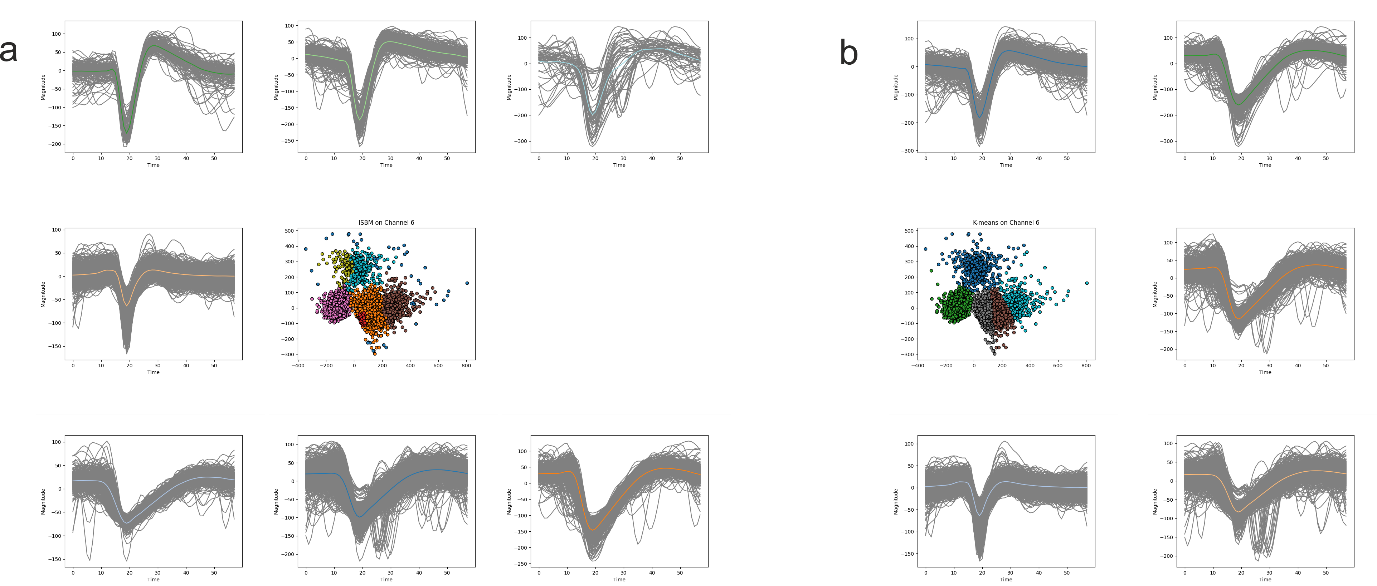


Figure 6 – Clustering of real data and the visualization of the generated waveforms (a) the clustering of ISBM encircled by the waveforms of the clusters formed, the colored line indicates which cluster it represents, and it is the average spike of that cluster (b) the clustering of K-Means (using k=5) encircled by the clusters formed.

1. Parametrization of each clustering algorithm for each dataset

|  | K-Means | DBSCAN | MeanShift | Agglom. | FCM | HDBSCAN | ISO-SPLIT | SBM | ISBM |
| --- | --- | --- | --- | --- | --- | --- | --- | --- | --- |
| UO | K=6 | Eps=0.5 | Q=0.07 | N=6 | C=6 | MCS=10 | - | PN=25 | PN=25 |
| Sim4 | K=5 | Eps=0.1 | Q=0.1 | N=5 | C=5 | MCS=10 | - | PN=10 | PN=10 |
| Sim1 | K=17 | Eps=0.05 | Q=0.05 | N=17 | C=17 | MCS=15 | - | PN=46 | PN=46 |
| Sim22 | K=7 | Eps=0.05 | Q=0.07 | N=7 | C=7 | MCS=30 | - | PN=30 | PN=46 |
| Sim21 | K=5 | Eps=0.1 | Q=0.1 | N=5 | C=5 | MCS=5 | - | PN=25 | PN=20 |
| Sim30 | K=6 | Eps=0.1 | Q=0.07 | N=6 | C=6 | MCS=50 | - | PN=30 | PN=40 |

1. Parametrization of each clustering algorithm for Varying number of dimensions of sim4

| Dimensions | KMeans | DBSCAN | MeanShift | Agglom. | FCM | HDBSCAN | ISO-SPLIT | SBM | ISBM |
| --- | --- | --- | --- | --- | --- | --- | --- | --- | --- |
| 2 | K=5 | e=.1 | Q=0.07 | N=5 | C=5 | MCS=10 | - | PN=10 | PN=10 |
| 3 | K=5 | e=.25 | Q=0.07 | N=5 | C=5 | MCS=30 | - | PN=12 | PN=12 |
| 4 | K=5 | e=.4 | Q=0.07 | N=5 | C=5 | MCS=30 | - | PN=8 | PN=8 |

1. Parametrization of each clustering algorithm for real data

| Data | KMeans | DBSCAN | MeanShift | Agglom. | FCM | SBM | ISBM |
| --- | --- | --- | --- | --- | --- | --- | --- |
| Tetrode Channel 1 | K=4 | e=18 | Q=0.7 | N=4 | C=4 | PN=10 | PN=20 |

# Appendix B

The first step of the algorithm is the normalization [5]. This step has been modified for the second improvement, the adaptive partitioning number.

1 normalisation(X, PN)

2 dim_var = variance(X, columns)

3 dim_var = dim_var / max_var

4 dim_var = dim_var * PN

5 normX = min_max(X)

6 normX = normX * dim_var

7 return normX

The second step of the algorithm is the chunkification. This step has been adapted for the new graph structure..

1 chunkification(X)

2 initialise graph

3 for point in X:

4 if point in graph

5 graph[point][count]+=1

6 else

7 graph.add(point, count=1, label=0, visited=0)

8 return graph

The third step of the algorithm is the identification of cluster centroid candidates [5]. This step has not been modified, with the exception of the data structure used.

1 findCentroids(graph,threshold)

2 clusterCentroids = []

3 for node in graph

4 if (node[count] >= threshold and isMaxima(graph, node))

5 add node to clusterCentroids

6 return clusterCentroids

Following the identification of cluster centroids, these are expanded sequentially. This only happens if the cluster centroid candidate has not been assigned to another cluster in the expansion of a previous cluster centroid.

The *expand* function has also been adapted for the graph structure, but its functionality remains the same. It receives as inputs the *graph*, the starting node (*start*) of the expansion, the current label (*clabel*) with which the current cluster is marked, and the list of cluster centres.

1 expand(graph,start,clabel,ccs)

2 expansionQueue = []

3 if graph[start][label]==0

4 expansionQueue.append(start)

5 graph[start][visited]=1

6 while expansionQueue not empty

7 node = expansionQueue.pop()

8 nbrs = getNeighbours(node)

9 for nbr in nbrs

10 distance = distance(start,nbr)

11 if not graph[nbr][visited] and graph[nbr][count]<=graph[node][count]

12 graph[nbr][visited] = 1

13 if graph[nbr][label] == 0

14 expansionQueue.push(nbr)

15 graph[nbr][label]=clabel

16 else //nbr was discovered already

17 expand = solveConflict(graph,node,nbr,ccs)

18 if expand == true

19 expansionQueue.push(nbr)

1 solveConflict(graph, node, currentCluster, oldCluster)

2 if node == oldCluster:

3 if graph[oldCluster][count] < graph[currentCluster][count]:

4 replace labels of oldCluster with currentCluster

5 else

6 replace labels of currentCluster with oldCluster

7 d1 = distance(node, currentCluster)

8 d2 = distance(node, oldCluster)

9 drop1 = calcDropoff(graph, currentCluster)

10 drop2 = calcDropoff(graph, oldCluster)

11 strength1= graph[currentCluster][count]/drop1 - drop1

12 strength2= graph[currentCluster][count]/drop2 - drop1

13 if strength > strength2

14 graph[node][label] = graph[currentCluster][label]

15 else

16 graph[node][label] = graph[oldCluster][label]

1 calcDropoff(graph, node)

2 nbrs = getNeighbours(node)

3 counts = 0

4 for nbr in nbrs:

5 counts += graph[nbr][count]

6 dropoff = graph[node][count] - mean(counts)

7 return dropoff

The *expand* function is based on the functionality of the BFS algorithm, it was chosen as such to expand to all the neighbours of a node first.

The calculation of the drop-off and the solving of conflicts [5] have been slightly changed from the original implementation. The drop-off is calculated as the mean of the counts of the neighbours of a node. The logic of the conflict solving function has not changed, only how the strength of a cluster is calculated.

The function *solveConflict* [5] intervenes in the case when the node towards which the expansion goes has already been assigned. The solution of the conflict can be either to merge the cluster and assimilate all of its nodes or to stop the expansion.

The last step, called “dechunkification” starts once the cluster candidates have been expanded. Each sample of the original dataset receives the label of the node it was assigned to.

1 dechunkification (graph, X)

2 initialise labels as empty list

3 for point in X:

4 labels.append(graph[point][label])

5 return pointLabels

*Labels* is an array that has the length equal to the number of samples in the dataset.
